# Homemade Filament Incorporating an Organic Modifier (Coumarin-Benzothiazole) for the Fabrication of High-Performance Voltammetric Sensors for Serotonin Determination

**DOI:** 10.1021/acsomega.6c06229

**Published:** 2026-07-23

**Authors:** Natália M. Caldas, Raíssa G. Gouvea, Yuri P. V. de Carvalho, Amanda G. Batista, Mikaelly O. B. de Sousa, Celia M. Ronconi, Lucas V. de Faria, Diego P. Rocha, Luana da S. M. Forezi, Rafael M. Dornellas

**Affiliations:** † Department of Analytical Chemistry, Institute of Chemistry, Fluminense Federal University, Niterói 24020-141, RJ, Brazil; ‡ Department of Organic Chemistry, Institute of Chemistry, Fluminense Federal University, Niterói 24020-141, RJ, Brazil; § Department of Inorganic Chemistry, Institute of Chemistry, Federal Fluminense University, Niterói 24020-141, RJ, Brazil; ∥ Institute of Chemistry, Federal University of Rio de Janeiro, Rio de Janeiro 21941-909, RJ, Brazil; ⊥ Department of Chemistry, Federal Institute of Paraná, Pitanga 85201-106, PR, Brazil

## Abstract

3D printing enables low-cost, high-performance electrochemical sensors for bioanalytical and clinical use. Herein, we report, for the first time, a graphite/polylactic acid (G/PLA) conductive composite modified with a coumarin–benzothiazole derivative for 3D-printed electrodes targeting enhanced serotonin detection. The optimized composition (G/coumarin-benzothiazole/PLA, 35:5:60 wt %) was processed with a 3D pen to produce conductive electrodes without electrochemical activation. Electrochemical impedance spectroscopy showed an ≈74% decrease in charge-transfer resistance for the modified material, while cyclic voltammetry revealed an ≈137% increase in serotonin oxidation current and a distinct oxidation peak at +0.375 V vs Ag|AgCl|KCl_(sat.)_. Optimized differential pulse voltammetry (modulation amplitude 70 mV; scan rate 30 mV s^–1^; step potential 8 mV) provided a linear range of 0.1–6.0 μmol L^–1^ (*R* ^2^ = 0.997), LOD = 0.075 μmol L^–1^, LOQ = 0.10 μmol L^–1^, and precision RSD < 5%. The sensor exhibited selectivity against common interferents (glucose, urea, uric acid, tyrosine, epinephrine, and dopamine), delivered near-quantitative recoveries in fortified human serum and cerebrospinal fluid, and interelectrode reproducibility RSD < 3%. To our knowledge, this G/coumarin-benzothiazole/PLA composite is the first 3D-printable, organic-modified conductive material demonstrating superior electrochemical performance, offering a robust, scalable platform for portable serotonin monitoring in bioanalytical and clinical applications.

## Graphical abstract

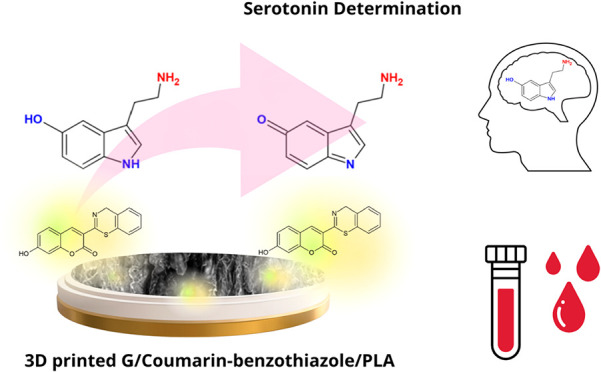

## 1 Introduction

Three-dimensional (3D) printing technology has democratized several areas of technology and scientific research.^1^ One of its main advantages lies in the relatively low-cost, simple, and accessible infrastructure required, which enables research fields with limited financial resources to conduct high-quality science.^2^ In this context, the main limitation shifts to the researcher’s ability to conceive, represent, and manipulate ideas, concepts, and structures that are not immediately present in the physical environment. Consequently, 3D printing stands out as a technology distinguished by its remarkable design freedom, scalability, versatility, and on-demand manufacturing capabilities.^1^ Owing to these advantages, the field of electroanalysis has greatly benefited from the integration of 3D printing technologies.^3,4^ Several strategies, including the fabrication of electrochemical cells, devices, and sensors, have been reported, demonstrating the successful integration of electroanalytical techniques with additive manufacturing.^4,5^ Nevertheless, some limitations remain, such as the discontinuation of commercially available conductive filaments, the relatively low content of conductive material in these filaments, and the need for postprocessing steps that can be time-consuming and may involve toxic solvents.^6–8^

In this context, a practical strategy to overcome these drawbacks is the in-house production of tailor-made conductive filaments.^7^ This approach allows for the incorporation of higher amounts of conductive fillers into the thermoplastic matrix, often eliminating the need for surface post-treatments.^7^ Furthermore, it enables fine control over material composition, facilitating the design of composite filaments with synergistic properties derived from different components, such as carbon-based materials, metallic (nano/micro)­particles, semiconductors, and two-dimensional materials.^3,4,7^ Despite these advances, to the best of our knowledge, no studies have reported the incorporation of functional organic modifiers into filaments to fabricate enhanced electrochemical sensors via 3D printing. Previous studies have described the addition of plasticizers (typically long-chain organic oils) into polymeric matrices; however, these additives mainly act as printability enhancers and do not contribute to improving interactions between the electrode surface and the target analyte.^9,10^

In this regard, coumarin and its derivatives emerge as promising, accessible, and structurally versatile organic compounds, which exhibit rich electronic properties and the ability to promote favorable interfacial interactions with electroactive species. Coumarins constitute a broad class of heterocyclic compounds characterized by a benzopyran-2-one core and are widely found in plants, fungi, and microorganisms, with more than 1300 naturally occurring compounds reported.^11^ Their excellent photophysical properties, including strong fluorescence, high quantum yields, visible-light excitation, and large Stokes shifts, have attracted considerable attention for sensing applications.^12,13^ In addition, the benzopyran-2-one scaffold can be readily functionalized at multiple positions, allowing the rational design of derivatives with enhanced selectivity and sensitivity toward specific analytes.^14–16^ Beyond their well-documented biological activities, such as antibacterial,^17^ antibiotic,^18^ antifungal,^19^ anticancer,^20^ antioxidant,^21^ anticoagulant,^22^ and neuroprotective^23^ effects, these structural and electronic features make coumarin-based compounds particularly attractive as functional modifiers in sensing platforms. Nevertheless, their integration into conductive filaments for additive manufacturing remains unexplored, highlighting an important opportunity to develop next-generation 3D-printed electrochemical sensors with enhanced analytical performance.

In this work, we report the development of a novel 3D-printable composite filament comprising coumarin-benzothiazole, graphite, and polylactic acid (PLA) (Figure 1). The incorporation of the coumarin–benzothiazole derivative into the PLA matrix, combined with the conductive properties of graphite (G), is intended to enhance the structural, thermal, electrical, and functional performance of the resulting composite. The developed material integrates the processability and biodegradability of PLA^24^ with the photophysical and molecular recognition properties of coumarin-based compounds^15^ and the electrical conductivity of graphite,^25^ resulting in a multifunctional composite suitable for advanced additive manufacturing applications. As a proof-of-concept, the fabricated sensor was used to detect the neurotransmitter serotonin (5-HT) with high sensitivity and selectivity in human serum and cerebrospinal fluid samples. These results demonstrate the potential of the proposed material for the fabrication of functional electrochemical devices and structurally robust components with tunable electronic and chemical properties.

**Figure 1:**
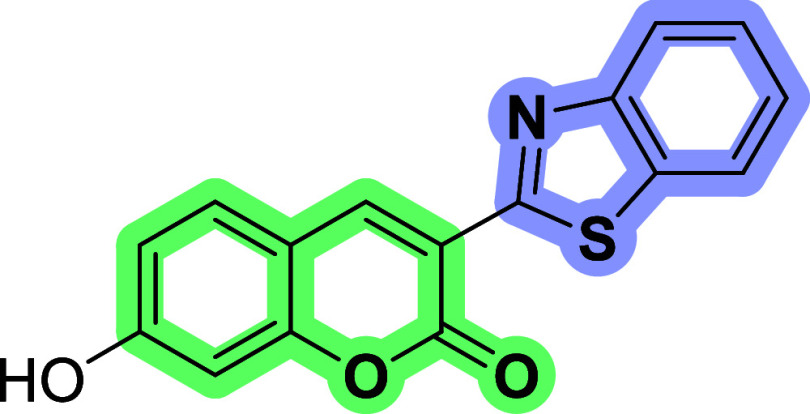
Chemical structure of the coumarin–benzothiazole derivative investigated in this study.

## 2 Experimental Section

### 2.1 Instrumentation

All electrochemical experiments were carried out in a conventional three-electrode configuration, with the 3D-G/PLA and 3D-G/coumarin-benzothiazole/PLA electrodes as working electrodes, an Ag|AgCl|KCl_(sat.)_ electrode as the reference, and a platinum wire as the counter electrode. The electrochemical cell was connected to a CompactStat potentiostat/galvanostat (Ivium Technologies, Eindhoven, The Netherlands) and operated through IviumSoft software, enabling accurate control and acquisition of the electrochemical signals. The measurements were performed in a glass electrochemical cell with a total volume of 10 mL.

Attenuated Total Reflectance Fourier-Transform Infrared (ATR-FTIR) spectra were collected over 550–4000 cm^–1^ using a Bruker α II spectrophotometer equipped with a ZnSe ATR crystal. X-ray powder diffraction (XRD) patterns were collected using a Rigaku MiniFlex II diffractometer with Cu Kα radiation (λ = 1.5406 Å), operating at 30 kV and 15 mA. Data were acquired over a 2θ range of 5–90 °, with a step size of 0.05° and a scan rate of 2°/min. The Raman spectra were measured using a Witec Alpha300 AR confocal Raman microscope with a 532 nm excitation wavelength (600 lines/mm diffraction grating, 0.5 mW power). The D and G bands were fitted with a Gaussian function in *OriginPro* 9.3 software. Scanning Electron Microscopy (SEM) was performed in a FEI Magellan 400 equipped with a field emission gun (FEG) (Hillsboro, USA). SEM images were acquired at an accelerating voltage of 2 kV with a current of 0.10 nA.

### 2.2 Reagents and Solutions

Deionized water (18.1 MΩ·cm) was produced using a Sartorius Arium Pro water purification system (Göttingen, Germany) and used throughout the preparation of all stock and working solutions. All chemicals employed in this work were of analytical grade and used as received, without further purification. 5-HT (99.0 wt. %), G powder (20 μm, 98.0 wt. %), glucose (GLU, 99.0 wt. %), dopamine (DP, 99.5 wt. %), uric acid (UA, 99.0 wt. %), epinephrine (EPI, 99.0 wt. %), tyrosine (TYR, 99.0 wt. %), and acetic acid (99.9% v/v) were purchased from Sigma-Aldrich (St. Louis, USA). Chloroform (99.8% v/v) was obtained from Synth (Diadema, Brazil) and boric acid (99.0 wt. %) from Panreac Química (Barcelona, Spain). Sodium chloride (NaCl, 99.0 wt. %) and potassium chloride (KCl, 99.5 wt. %) were supplied by Merck (Darmstadt, Germany), while ferricyanide (99.0 wt. %), calcium chloride (CaCl_2_, 98.0 wt. %), ferrocyanide (99.0 wt. %), sodium bicarbonate (NaHCO_3_, 98.0 wt. %), and urea (98.0 wt.%) were supplied by Vetec (Duque de Caxias, Brazil). The ABS filament was sourced from GTMax (São Paulo, Brazil), and PLA pellets were provided by 3DLAB (Minas Gerais, Brazil). Phosphoric acid (85.0% v/v) was obtained from Hexis (São Paulo, Brazil).

The electrochemical analyses were performed using a 0.12 mol L^–1^ phosphate buffer (pH 7.0) as the supporting electrolyte, while the effect of pH on the oxidation of 5-HT was examined with Britton-Robinson (BR) buffer (0.12 mol L^–1^, pH 5.0–10.0). The latter solution is composed of an equimolar mixture (0.04 mol L^–1^) of boric, phosphoric, and acetic acids.

### 2.3 Design and Fabrication of Carbon–Coumarin-Benzothiazole-Based Conductive Filaments for 3D-Printed Electrochemical Sensing Platforms

The flexibility and mechanical robustness of the fabricated filaments were governed by the PLA content, which was adjusted to proportions that ensured reliable 3D printing performance. In all formulations, the PLA fraction was maintained above the minimum threshold required to preserve printability, as previously reported in the literature.^26–28^ Based on this criterion, the following compositions were investigated: (i) G/PLA (30:70 wt. %, control), (ii) G/coumarin-benzothiazole/PLA (30:2.5:67.5 wt. %), (iii) G/coumarin-benzothiazole/PLA (30:5:65 wt. %), and (iv) G/coumarin-benzothiazole/PLA (30:7.5:62.5 wt. %).

PLA was initially dissolved in a chloroform/acetone mixture (1:3 v/v) under reflux, at 110 °C with vigorous mechanical stirring (5000 rpm) for 1 h to ensure complete dissolution. A presonicated G/coumarin-benzothiazole dispersion, prepared in the same solvent system (20 min sonication), was then gradually incorporated into the polymer solution. The system was maintained under reflux with continuous stirring for an additional 2 h to promote homogeneous physical dispersion of the G/coumarin-benzothiazole phase within the PLA matrix, without any chemical reaction between the components. The above-mentioned procedure was performed inside a fume hood.

The resulting suspension was cast onto parchment paper and dried at room temperature (25 °C) for 24 h to allow complete solvent evaporation. The dried material was subsequently cut into approximately 1 cm^2^ fragments and processed using a Filmaq 3D filament extruder (Curitiba, Brazil) at 190 °C and maximum screw speed. The extruded filaments (1.75 mm diameter) were collected and stored in sealed plastic bags under ambient conditions to minimize moisture uptake. Precisely engineered cylindrical architectures were developed in SolidWorks computer-aided design software and fabricated via fused deposition modeling (FDM) on a Creality K1 3D printer (Shenzhen, China) using acrylonitrile butadiene styrene (ABS) filament. The printing parameters were carefully selected to provide structural stability and reproducibility, using a 0.4 mm nozzle, an extrusion temperature of 230 °C, a heated bed temperature of 110 °C, a printing speed of 100 mm s^–1^, a layer height of 0.3 mm, three perimeter shells, and a 100% infill density. The resulting ABS structures served as consistent, reliable substrates for subsequent electrode assembly.

After fabrication, a copper wire was embedded in each printed cylinder to provide a stable and low-resistance electrical connection to the potentiostat. The conductive composite filament was then selectively deposited onto a predefined geometric area (0.196 cm^2^) using a Sanmersen 3D printing pen (Shenzhen, China) operated at 190 °C, enabling controlled material placement and effective integration with the supporting body. To promote surface homogeneity and consistent electrochemical performance, the electrodes were subjected to a standardized wet-polishing procedure using progressively finer abrasive papers (600- and 1200-grit). This finishing step improved surface uniformity and facilitated adequate exposure of the conductive phase. A schematic overview of the electrode fabrication workflow is presented in Figure 2.

**Figure 2:**
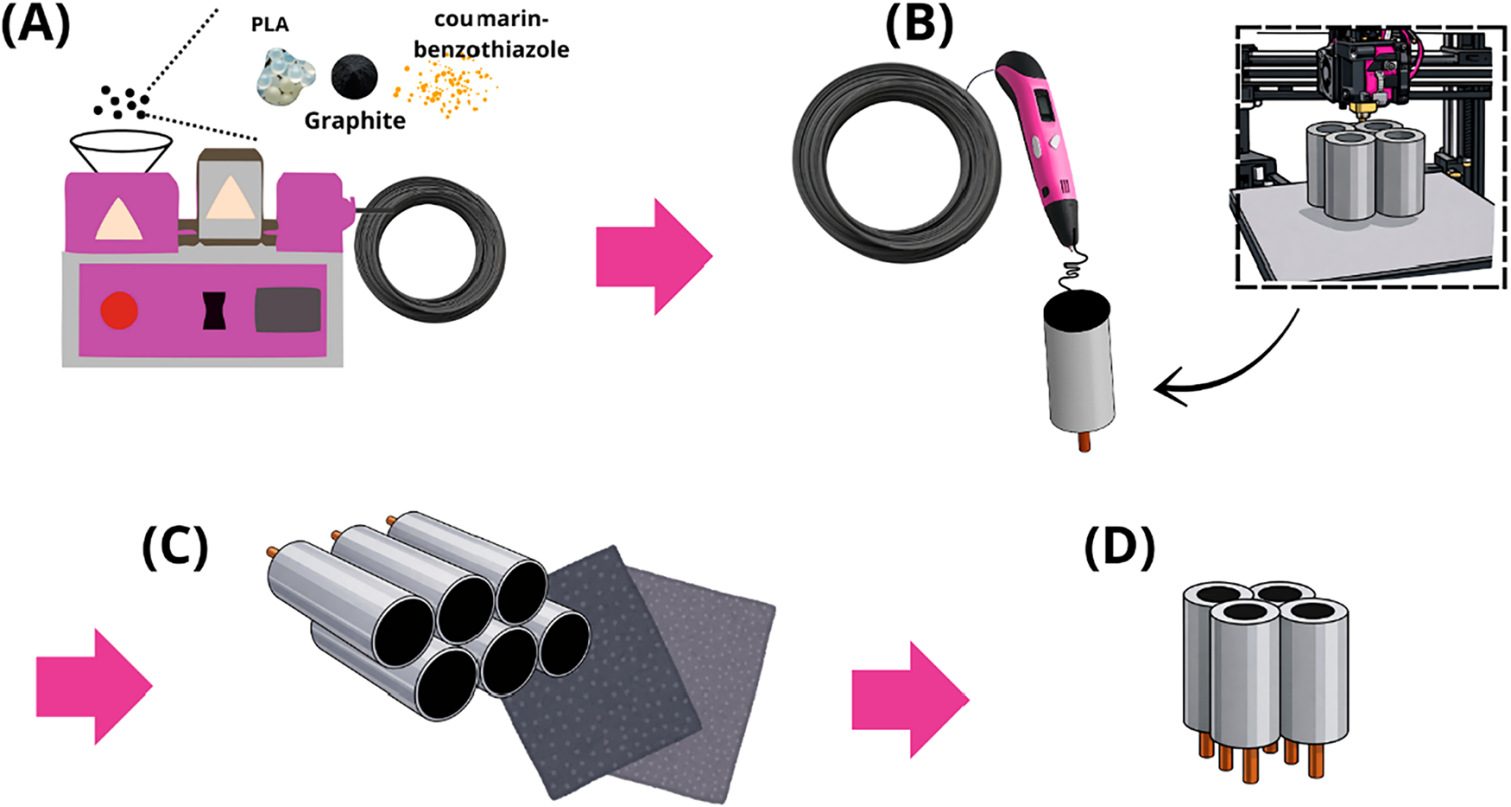
Schematic illustration of the fabrication stages of the G/coumarin-benzothiazole/PLA electrode. (A) Production of the G/coumarin-benzothiazole/PLA filament via extrusion; (B) fabrication of the 3D-printed ABS-based cylindrical support for the working electrode, followed by controlled deposition of the conductive composite filament onto the printed substrate using a 3D printing pen; (C) surface finishing by sequential polishing with sandpapers of progressively finer grit sizes; and (D) final ready-to-use 3D-printed electrode. Created by the authors using Canva.

### 2.4 Sample Preparation Procedure

Two artificial biological fluid matrices were selected to evaluate the analytical applicability of the proposed method: human serum obtained from Sigma-Aldrich (St. Louis, USA) and an artificial cerebrospinal fluid prepared according to the procedure reported by de Toledo and co-workers.^29^ Briefly, the artificial cerebrospinal fluid was synthetically prepared by dissolving 2.1 g of NaCl, 0.07 g of KCl, 0.08 g of CaCl_2_, 0.2 g of glucose, 0.4 g of NaHCO_3_, and 2.0 mg of urea in a 250 mL volumetric flask containing ultrapure water. After complete dissolution of all components, the pH of the solution was carefully adjusted to 7.4 with a dilute base to simulate physiological conditions. The solution was used immediately after preparation to minimize possible urea hydrolysis and compositional changes. Before electrochemical measurements, both human serum and artificial cerebrospinal fluid samples were subjected to a simple dilution step in the supporting electrolyte (1:10 v/v), without additional pretreatment, filtration, or extraction. This straightforward protocol was adopted to demonstrate the practical feasibility of the proposed sensor for direct analysis in complex matrices. Method accuracy was assessed using spike-and-recovery experiments at two 5-HT concentrations, 0.5 and 1.0 μmol L^–1^.

## 3 Results and Discussion

### 3.1 Structural, Morphological, and Electrochemical Evaluation of the Fabricated Materials

The ATR-FTIR spectrum of pure coumarin-benzothiazole (Figure 3 (dark yellow)) exhibits the characteristic vibrational features of the coumarin-benzothiazole framework. A broad band centered at ∼3350 cm^–1^ is assigned to the phenolic O–H stretching vibration. Aromatic C–H stretching is observed at ∼3068 cm^–1^. The strong absorption at 1722 cm^–1^ corresponds to the lactone C=O stretching mode of the coumarin ring.^30,31^ In the 1650–1500 cm^–1^ region, coupled skeletal vibrations of the conjugated system are observed, with the band at ∼1606 cm^–1^ mainly attributed to aromatic C=C stretching and the band at ∼1574 cm^–1^ predominantly assigned to the C=N stretching vibration of the benzothiazole moiety.^32^ Additional features at 1311 and 1206–1129 cm^–1^ are associated with C–O and C–O–C stretching modes of the phenolic and lactone functionalities,^33,34^ while the band near 757 cm^–1^ is attributed to vibrations involving the benzothiazole C–S bond,^31^ with possible contribution from out-of-plane aromatic C–H bending.

**Figure 3:**
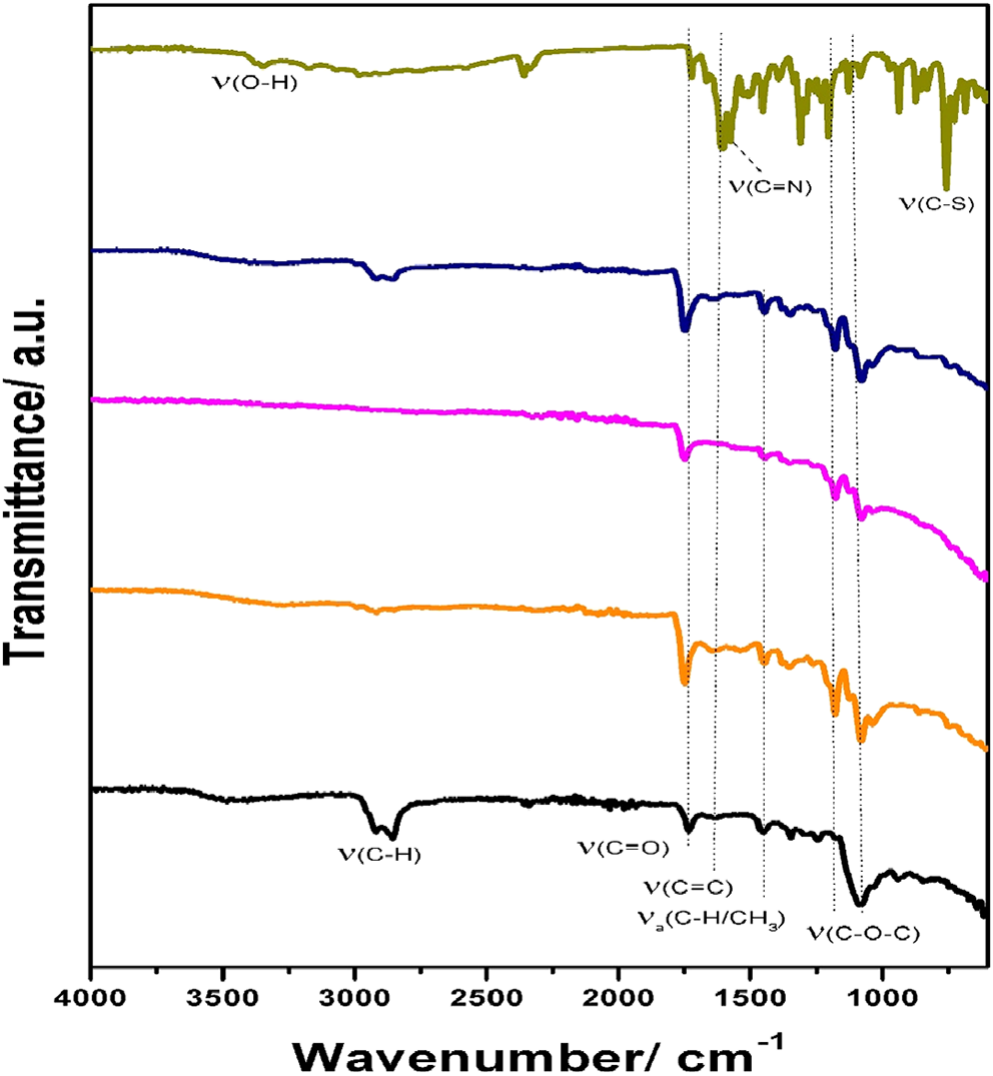
ATR-FTIR spectra of coumarin-benzothiazole (dark yellow), G/PLA (black line), G/coumarin-benzothiazole/PLA (30:2.5:67.5 wt. %, orange line), G/coumarin-benzothiazole/PLA (30:5:65 wt. %, magenta line), and G/coumarin-benzothiazole/PLA (30:7.5:62.5 wt. %, dark blue line).

The ATR-FTIR spectra of the G/coumarin-benzothiazole/PLA electrodes display the characteristic vibrational features of PLA, indicating that the polymer structure is preserved across all formulations, regardless of coumarin content (Figure 3). In all samples, with the exception of the composite containing 5 wt. % coumarin-benzothiazole, where several bands appear less defined, the C–H stretching region (≈2920–2850 cm^–1^) is evident, along with the intense ester C=O stretching band at 1747–1733 cm^–1^, consistent with values reported for PLA-based materials.^35–37^ In the fingerprint region, the spectra display the expected ester ν­(C–O–C) stretching modes between 1180–1080 cm^–1^, as well as the CH_3_ rocking and C–CH_3_ vibrations near 1125 and 1038–1040 cm^–1^. Bending modes of CH and CH_3_ groups are also observed in the 1450–1350 cm^–1^ range.^35^ Weak contributions between 1640–1540 cm^–1^ correspond to C=C/C–C stretching of the graphitic phase^37^ although their intensity is low and remains largely unchanged across the formulations. Distinct coumarin-benzothiazole-related absorptions are not resolved, as its main aromatic and carbonyl modes overlap with the dominant PLA and graphite vibrations.^38^ Overall, the FTIR profiles of the composites remain similar, indicating that the addition of coumarin does not introduce detectable changes to the vibrational signatures of the composites. The main absorption bands identified in the G/PLA and G/coumarin-benzothiazole/PLA electrodes and their corresponding vibrational assignments are displayed in Table S1.

To investigate potential structural changes induced by material modifications, XRD analysis was performed as shown in Figure S1. All electrodes exhibit a broad peak at 2q ≈ 20°, associated with the polymer’s amorphous fraction.^39^ A sharp peak at 2q ≈ 26°, corresponding to the (002) planes of multilayer graphite with an interlayer spacing of ∼3.36 Å, is also present in all formulations, confirming that the processing does not fully exfoliate the graphitic domains.^36,40,41^ Notably, the intensity and slight shifts of this peak vary with coumarin-benzothiazole content, suggesting modest changes in stacking order or microstrain within the graphitic phase. Additional graphitic reflections (101, 004, 110, 112)^42^ are preserved, and the absence of new diffraction peaks indicates that no secondary crystalline phases are formed, demonstrating that the PLA–graphite framework accommodates coumarin-benzothiazole without significant structural disruption. Table S2 presents the XRD peak parameters corresponding to the graphitic (002) reflection in G/PLA and coumarin-benzothiazole-based electrodes.

The Raman spectra of the coumarin-benzothiazole-containing electrodes exhibited a pronounced fluorescence background arising from the chromophore. To ensure reliable band identification, all spectra were baseline-corrected, a procedure that effectively removed the fluorescence contribution while preserving peak positions and relative intensities. After baseline correction, all electrodes display the characteristic vibrational features of graphitic materials (see Figure 4). The D band, observed at ∼1342–1346 cm^–1^, corresponds to the A_1_g breathing mode of sp2 carbon rings and becomes Raman-active in the presence of structural defects. The G band, located at ∼1575 cm^–1^, is assigned to the E_2_g in-plane stretching mode of sp^2^-hybridized carbons within the graphitic lattice.^43,44^ All electrodes also display the 2D band at ∼2700 cm^–1^, an overtone of the D band arising from a second-order scattering process.^43^ Its single-peak profile is consistent with a two-dimensional graphitic structure with imperfect three-dimensional stacking, as expected for multilayer graphite.^45^ The *I*_D_/*I*_G_ ratio decreases markedly upon addition of 2.5 and 5 wt. % coumarin-benzothiazole (from 0.528 (control electrode) to 0.253 and 0.230, respectively), indicating a reduction in defect-activated scattering. This trend indicates that low coumarin-benzothiazole concentrations fill or passivate defect sites, yielding a more ordered effective sp2 network.^46^ However, when the coumarin-benzothiazole content is increased to 7.5 wt. %, the *I*_D_/*I*_G_ ratio rises again (0.368), implying that excess coumarin introduces microstructural perturbations, such as local strain or partial disruption of the graphitic stacking, that enhance defect density, in agreement with the established correlation between higher *I*_D_/*I*_G_ and increased disorder in graphitic materials.^47–49^ Table S3 details the positions of the D and G bands and the *I*_D_/*I*_G_ ratio obtained for the different electrodes.

**Figure 4:**
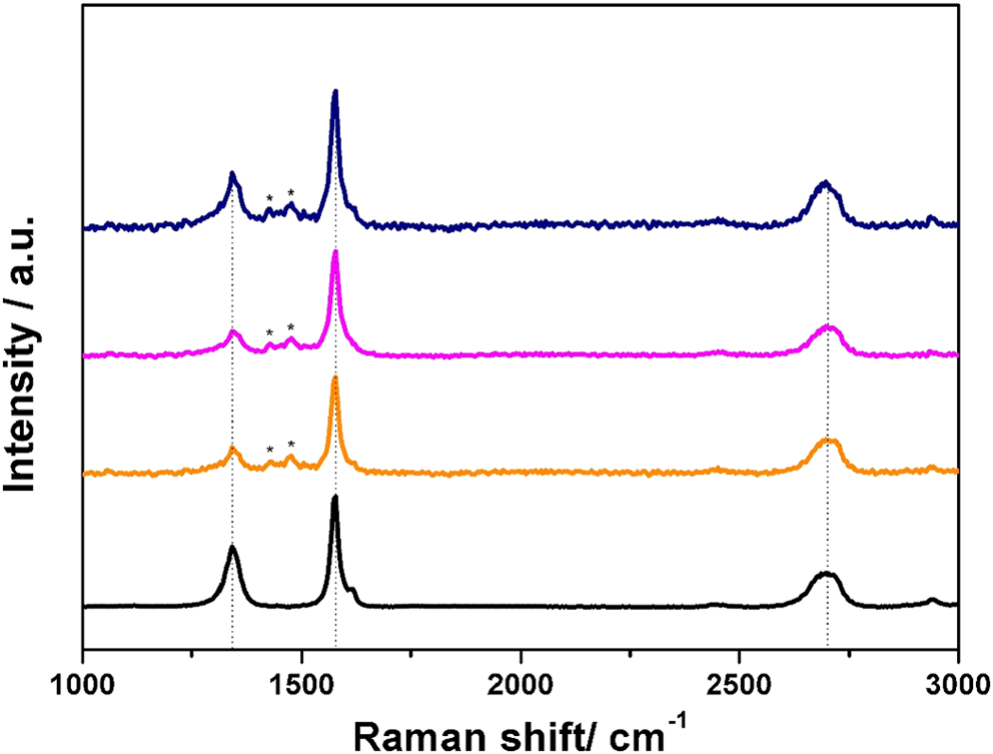
Raman spectra of the G/PLA (30:70 wt. %, black line), G/coumarin-benzothiazole/PLA (30:2.5:67.5 wt. %, orange line), G/coumarin-benzothiazole/PLA (30:5:65 wt. %, magenta line), and G/coumarin-benzothiazole/PLA (30:7.5:62.5 wt. %, dark blue line).

In addition to the characteristic carbon bands, the coumarin-benzothiazole-containing electrodes show weak and broad contributions at around 1425 and 1475 cm^–1^, which are absent in the G/PLA electrode. These signals fall within the spectral region typically assigned to aromatic ring stretching, C=C stretching, and in-plane CH bending modes of coumarin derivatives, as reported in Raman studies of coumarin powders and thin films.^50–52^ Their presence confirms that the chromophore remains incorporated and spectroscopically active within the composite and suggests a degree of interaction with the graphitic domains, likely mediated by π–π stacking, a well-documented supramolecular interaction between aromatic molecules and sp^2^-carbon surfaces.^53^

Considering the morphological aspect, the SEM images show that the G/PLA (30:70 wt. %) electrode exhibits a densely packed morphology, with graphite platelets arranged in a compact, layered structure. In contrast, all coumarin-benzothiazole-containing electrodes display a noticeably more open and irregular surface, marked by increased roughness and the presence of voids between carbon domains. Although the general appearance of the 2.5, 5.0, and 7.5 wt. % samples are similar; they consistently exhibit a less cohesive microstructure than the G/PLA (30:70 wt. %), indicating that coumarin-benzothiazole disrupts the packing of graphite within the PLA matrix, as shown in Figure 5.

**Figure 5:**
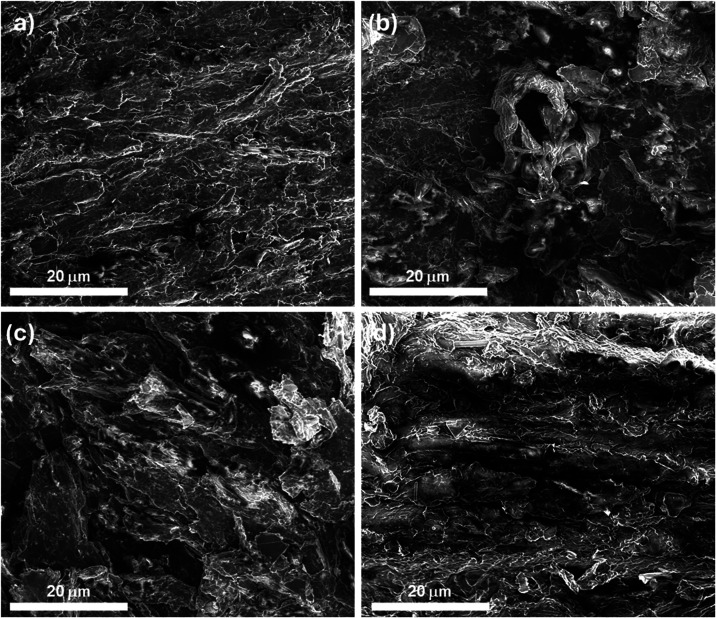
SEM images recorded at a 5000× magnification of (a) G/PLA (30:70 wt. %) and G/coumarin-benzothiazole/PLA electrodes at (b) 30:2.5:67.5 wt. %, (c) 30:5:65 wt. %, and (d) 30:7.5:62.5 wt. %.

Because coumarin derivatives are known to exhibit anodic electrochemical activity,^54,55^ voltammetric measurements from 0.0 to +1.5 V were executed in the presence of supporting electrolyte (0.1 mol L^–1^ KCl) to verify the incorporation of coumarin-benzothiazole onto the surface of the proposed electrodes, as shown in Figure S2. Using the control electrode (G/PLA), no faradaic processes were observed, indicating that coumarin-benzothiazole was absent from the surface. On the other hand, the electrodes loading 2.5, 5.0, and 7.5 wt. % coumarin-benzothiazole exhibited an irreversible oxidation process around +1.0 V, in agreement with a previous report.^55^ Interestingly, as the coumarin-benzothiazole content increased, a higher peak current was observed, suggesting that coumarin-benzothiazole was successfully incorporated in different proportions within the G/PLA matrix, which is in accordance with Raman, ATR-FTIR, and SEM analysis.

### 3.2 Explorataory Electrochemical Studies on G/PLA and G/coumarin-Benzothiazole/PLA Electrodes

Initially, cyclic voltammetry (CV) analyses were conducted using 1.0 mmol L^–1^ [Fe­(CN)_6_]^4–^/^3–^, a well-known and widely used redox probe, in 0.1 mol L^–1^ KCl (supporting electrolyte) to evaluate the influence of coumarin-benzothiazole on the sensor’s electrochemical responses. As shown in Figure S3 and corresponding detailed electrochemical data in Table S4, increasing the coumarin-benzothiazole content up to 5 wt. % led to higher anodic (*I*_pa_) and cathodic (*I*_pc_) peak currents, along with a decrease in peak-to-peak separation (Δ*E*), indicating a marked improvement in the electrochemical behavior of the redox probe. Compared with the control electrode (coumarin-benzothiazole-free), the 5 wt. % coumarin-benzothiazole-loaded electrode exhibited an increase in *I*_pa_ from 9.5 to 22.5 μA, as well as a decrease in Δ*E* from 0.535 to 0.160 V. The inferior performance of the 7.5 wt. % coumarin-benzothiazole electrode relative to the 5 wt. % formulation is likely due to disruption of graphite packing within the thermoplastic matrix, as revealed by SEM images. At this higher coumarin-benzothiazole-loading, the availability of conductive graphite pathways may have been compromised, negatively affecting the electrodés electrochemical performance. Therefore, a more efficient synergistic interaction between graphite and coumarin-benzothiazole was achieved for the G/coumarin-benzothiazole/PLA (30:5:65 wt. %) electrode, and, accordingly, subsequent electrochemical experiments were carried out using this composition, compared with the control electrode (G/PLA, 30:70 wt. %).

Information on the charge-transfer resistance (*R*_ct_) of the redox probe at different electrode/solution interfaces was obtained using electrochemical impedance spectroscopy (EIS). In both cases, the Nyquist plots showed well-defined semicircles bounded at high frequencies by the solution resistance (*R*_s_) and at low frequencies by a diffusion-controlled mass-transport region (45° linear segment) associated with the Warburg element^56^ (Figure 6A). The inset of Figure 6A shows the Randles equivalent circuit, which adequately represents the electrical behavior of the investigated electrochemical system. In addition to the components described above, a constant phase element (CPE) was included to account for the capacitive contribution of the electrical double layer. Notably, the G/coumarin-benzothiazole/PLA (30:5:65 wt. %) electrode exhibited a substantially smaller semicircle than the control electrode, resulting in Rct values of 1.85 and 7.07 KΩ, respectively. This demonstrates enhanced charge-transfer properties in the presence of coumarin-benzothiazole. A reasonable rationale for the improved charge-transfer kinetics is the higher density of electroactive sites introduced by the coumarin-benzothiazole moiety, attributable to its conjugated aromatic system and oxygen-containing functional groups. Following, the electrochemically active surface areas of the G/PLA and G/coumarin-benzothiazole/PLA (5 wt. % coumarin-benzothiazole) electrodes were therefore estimated following the Randles–Ševčík recommendation.^57,58^ CV was performed in 1 mmol L^–1^ [Fe­(CN)_6_]^4–/3–^ dissolved in 0.1 mol L^–1^ KCl at scan rates ranging from 10 to 100 mV s^–1^ (Figure S4A). As depicted in Figure S4B, the linear dependence of *I*_p_ on the square root of scan rate (ν^1/2^) indicates diffusion-controlled redox behavior at both electrode surfaces. The calculated electroactive areas were 1.34 and 4.75 mm^2^ for G/PLA and G/coumarin-benzothiazole/PLA (5 wt. % coumarin-benzothiazole), respectively, underscoring the favorable electrical contribution of coumarin-benzothiazole to the sensor architecture.

**Figure 6:**
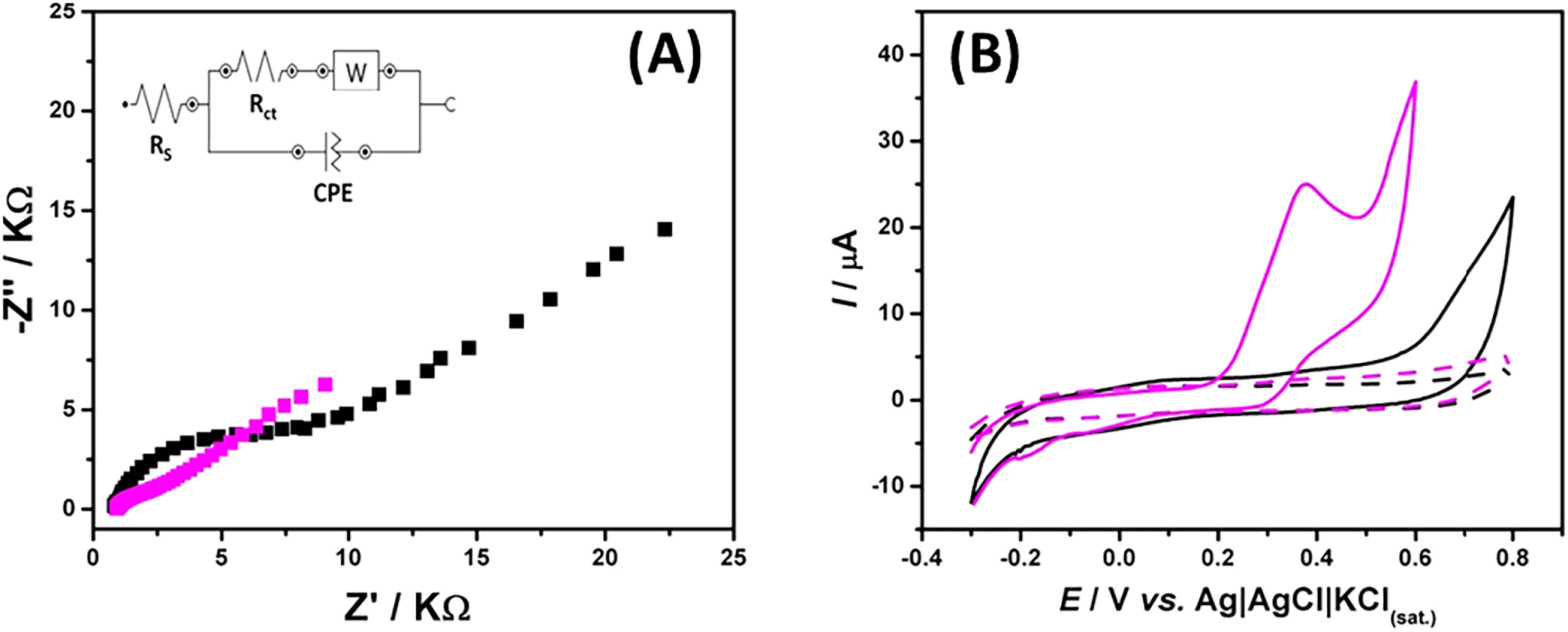
(A) Nyquist plot from EIS spectra acquired for 3D-printed G/PLA (black square) and G/coumarin-benzothiazole/PLA (magenta square) electrode surfaces. The inset represents the corresponding Randles equivalent circuit. (B) Electrochemical responses were recorded using 0.5 mmol L^–1^ of 5-HT in 0.12 mol L^–1^ phosphate buffer (pH 7.0) solution on the G/PLA (30:70 wt. %, black line) and G/coumarin-benzothiazole/PLA (30:5:65 wt. %, magenta line). The dashed lines indicate the respective blanks. EIS conditions: frequency between 1000 and 0.1 Hz, 10 data points per decade, and 10 mV. CVs conditions: step potential of 5 mV and scan rate of 50 mV s^–1^.

After elucidating the electrochemical behavior of both electrodes with the redox probe, attention was turned to the target analyte, 5-HT, a biologically significant neurotransmitter (Figure 6B). Voltammetric measurements performed in 0.5 mmol L^–1^ 5-HT dissolved in 0.12 mol L^–1^ phosphate buffer (pH 7.0) revealed a single irreversible oxidation process at both electrode surfaces. At the control electrode (G/PLA), oxidation occurred at approximately +0.68 V vs Ag|AgCl|KCl_(sat.)_, with an *I*_p_ of 4.71 μA. In striking contrast, the coumarin-benzothiazole-incorporating electrode exhibited the same process at a substantially lower potential, around +0.34 V vs Ag|AgCl|KCl_(sat.)_, along with a markedly enhanced *I*_p_ of 19.73 μA. These results reveal a substantial 4.2-fold amplification of the electrochemical signal for 5-HT, accompanied by a significant 0.34 V cathodic shift in the oxidation potential. Such simultaneous gains in analytical response and reduced overpotential indicate an increased density of active sites and suggest a possible electrocatalytic contribution from coumarin-benzothiazole, which may facilitate faster electron-transfer kinetics. Collectively, these features highlight the improved analytical performance of the coumarin-benzothiazole-based electrode and its potential as an effective platform for neurotransmitter detection.

For large-scale electrode production, between-device reproducibility is a critical parameter. To assess this, three independent electrodes were produced from different segments of conductive filaments containing 5 wt. % coumarin-benzothiazole. The electrochemical response toward the [Fe­(CN)_6_]^4–/3–^, commonly employed for this purpose, was used as the analytical metric (see Figure S5). A relative standard deviation (RSD) of 2.6% was obtained considering the anodic *I*_p_, indicating excellent repeatability of the proposed manufacturing procedure and supporting its suitability for the consistent production of low-cost electrochemical sensors.

### 3.3 Electrochemical Behavior of 5-HT at G/coumarin-Benzothiazole/PLA Electrode Surfaces

The effect of supporting electrolyte pH on the electrochemical behavior of 5-HT was systematically assessed using DPV. In this study, a 0.12 mol L^–1^ BR buffer was employed as the supporting electrolyte owing to its broad and stable buffering capacity across an extended pH range. Measurements were conducted in solutions containing 4 μmol L^–1^ 5-HT over the pH range 5.0 to 10.0 (Figure 7A). As the solution pH increased, the *E*_p_ of 5-HT shifted toward less positive values and showed a linear correlation with pH (Figure 7B, magenta line). This trend is indicative of a proton-coupled electron-transfer mechanism, where the slope of −52.1 mV pH^–1^ supports the involvement of an equal number of protons and electrons in the electrochemical oxidation of 5-HT. Conversely, the *I*_p_ displayed a parabolic dependence on pH (Figure 7B, black line). The analytical signal increased progressively with rising pH, reaching a maximum at neutral conditions (pH 7.0) and subsequently declining at more alkaline conditions. Considering the combined influence of favorable *E*_p_, enhanced signal intensity, and pH conditions representative of physiological environments, pH 7.0 was selected as the optimal medium for further analyses.

**Figure 7:**
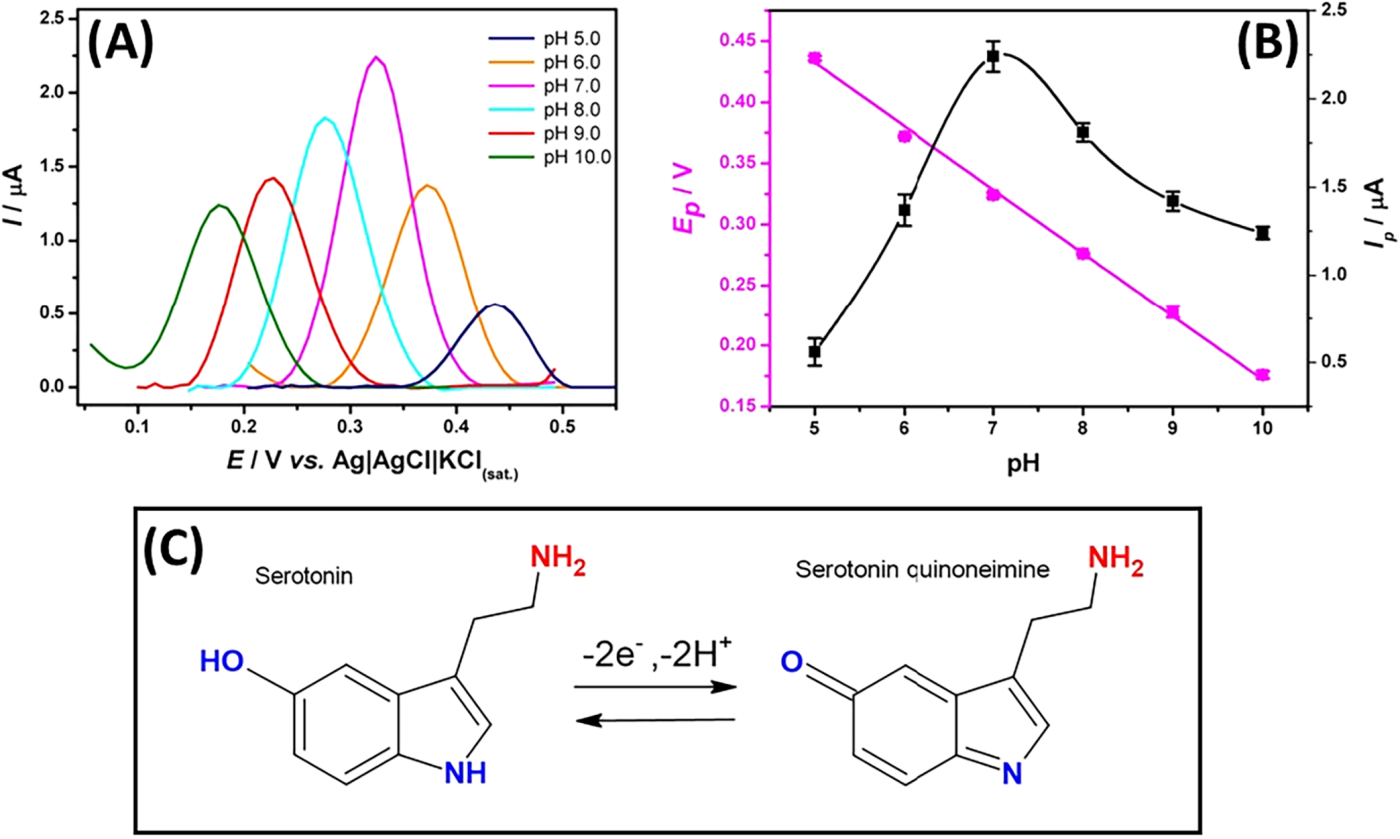
(A) DPV electrochemical response recordings in the presence of 4 μmol L^–1^ of 5-HT upon the 3D-printed G/coumarin-benzothiazole/PLA (35:5:60 wt. %) electrode at different pH values (5.0–10.0) using 0.12 mol L^–1^ BR buffer as background electrolyte. (B) Relationship between *E*_p_ and pH (magenta line) and between *I*_p_ and pH (black line). (C) Proposed mechanistic pathway for the electrochemical oxidation of 5-HT. DPV conditions: modulation amplitude of 70 mV, scan rate of 30 mV s^–1^, and step potential of 8 mV.

Understanding the analyte’s active site, responsible for its electrochemical response, is of considerable relevance. Accordingly, efforts were undertaken to propose a plausible oxidation mechanism for 5-HT at the proposed sensing platform. Initially, the influence of scan rate (10–80 mV s^–1^) on the oxidation process was examined (Figure S6A). A linear relationship between Log *I* and Log *v* was observed (Figure S6B), with a slope of 0.61, indicating that the rate-determining step is in a mixed-control regime with contributions from both diffusion and adsorption of 5-HT species. Furthermore, the scan rate also affected the peak potential of 5-HT, enabling a linear correlation between *E*_p_ and Log *v* to be established (Figure S7C). From this relationship, and following Laviron’s approach,^59^ the number of electrons involved in the process was estimated to be two. Therefore, the electrochemical oxidation of 5-HT involves the transfer of two electrons and two protons. Based on these findings, a mechanistic pathway was proposed involving the oxidation of the phenolic hydroxyl group to form a quinone-imine derivative, as illustrated in Figure 7C. This mechanistic route is consistent with that reported in previous studies.^60,61^

### 3.4 Optimization of a Voltammetric Method and Analytical Performance for 5-HT Sensing

The electrochemical response of 4 μmol L^–1^ 5-HT was evaluated using the two pulse voltammetric techniques most frequently reported in the literature, DPV and SWV.^62–64^ As shown in Figure S7, DPV delivered a more defined oxidation peak and an analytical signal approximately 3-fold higher than that obtained with SWV; consequently, DPV was selected for subsequent analyses. A similar behavior was also observed in a previous study employing a 3D-printed electrochemical platform.^62^ The instrumental parameters of DPV, modulation amplitude (10–90 mV), step potential (1–9 mV), and scan rate (10–50 mV s^–1^), were then optimized using a univariate approach with triplicate measurements (Figure S8). The selection criteria were to maximize peak current, improve peak definition, and minimize signal variability. Based on these considerations, the optimal conditions were a modulation amplitude of 70 mV, a scan rate of 30 mV s^–1^, and a step potential of 8 mV.

Under these conditions, calibration curves were constructed using both the control (G/PLA (30:70 wt. %)) and coumarin-benzothiazole-based (G/coumarin-benzothiazole/PLA (30:5:65 wt. %)) electrodes (Figure 8A–B). The control electrode exhibited a linear response over the concentration range of 0.75–3.0 μmol L^–1^, *R* ^2^ = 0.997 (Figure 8C, black line), whereas the coumarin-benzothiazole-based electrode showed a broader linear range of 0.1–6.0 μmol L^–1^, *R* ^2^ = 0.997 (Figure 8C, magenta line) and an 8.5-fold increase in sensitivity. Additional analytical performance parameters, including the limit of detection (LOD), limit of quantification (LOQ), and repeatability, were also assessed. LOD and LOQ values were calculated according to IUPAC recommendations,^65^ while repeatability (*n* = 8) was evaluated at two concentration levels (2.5 and 5.5 μmol L^–1^) (Figure S9). For the G/PLA and G/coumarin-benzothiazole/PLA (30:5:65 wt. %) electrodes, LODs of 0.16 and 0.075 μmol L^–1^ and LOQs of 1.0 and 0.10 μmol L^–1^, respectively, were obtained, demonstrating the coumarin-benzothiazole-based electrode’s capability to quantify 5-HT at submicromolar levels. Repeatability tests yielded RSD values below 4.53%, confirming the precision of the analyses when the proposed sensing-loaded-coumarin-benzothiazole platform was used. A comprehensive comparison of the analytical figures of merit for both sensors is presented in Table 1.

**Figure 8:**
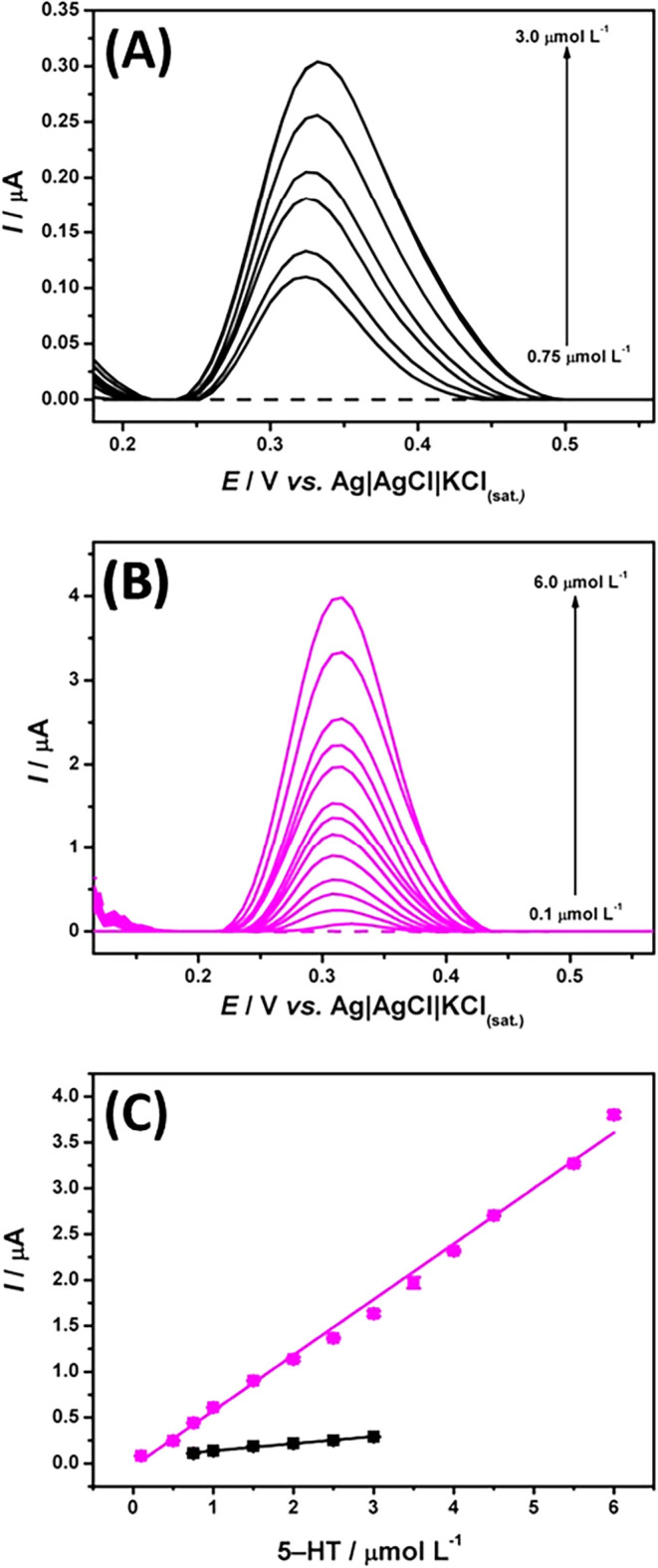
DPV electrochemical responses (*n* = 3) recorded using increasing levels of 5-HT on the (A) 3D-printed G/PLA (30:70 wt. %) (0.75 to 3.0 μmol L^–1^) and (B) 3D-printed G/coumarin-benzothiazole/PLA (30:5:65 wt. %) (0.1 to 6.0 μmol L^–1^) electrodes; (C) Respective calibration plots achieved using G/PLA (30:70 wt. %, black line) and G/coumarin-benzothiazole/PLA (30:5:65 wt. %) (magenta line) electrodes. DPV conditions: modulation amplitude of 70 mV, scan rate of 30 mV s^–1^, and step potential of 8 mV. Background electrolyte: 0.12 mol L^–1^ phosphate buffer (pH 7.0).

**Table 1:** Analytical Features Achieved towards 5-HT Sensing Using G/PLA (30:70 wt. %, Control Electrode) and G/coumarin-Benzothiazole/PLA (30:5:65 wt. %) Electrodes

Analytical features	G/PLA (30:70 wt. %)	G/coumarin-benzothiazole/PLA (30:5:65 wt. %)
Linear Range/μmol L^–1^	0.75–3.0	0.1–6.0
LOD/μmol L^–1^	0.16	0.075
LOQ/μmol L^–1^	0.75	0.1
Sensitivity/μmol^–1^ L μA	0.070	0.60
*R* ^2^	0.994	0.997

With a view toward applications in biological matrices, the selectivity of the proposed sensor was systematically evaluated in the presence of species commonly encountered in biological fluids, such as glucose, urea, uric acid, tyrosine, epinephrine, and dopamine, as recommended in a previous study.^62^ The potential interferents were investigated at a 3.2:1 concentration ratio relative to the analyte, as depicted in Figure S10. The experiments were performed using 2.5 μmol L^–1^ 5-HT and 8.0 μmol L^–1^ of each interfering species, over a potential range from +0.1 to +0.6 V vs Ag|AgCl|KCl_(sat.)_, corresponding to the region in which 5-HT exhibits its electrochemical response.

As shown in Figures S10A–D, glucose, urea, uric acid, and tyrosine did not yield any detectable electrochemical signal under the selected experimental conditions. Glucose and urea are not electroactive on carbon-based platforms and consequently did not produce any measurable response.^66^ Although uric acid and tyrosine are electroactive compounds, tyrosine typically undergoes oxidation at potentials close to +1.0 V vs Ag|AgCl|KCl_(sat.)_ on 3D-printed carbon-based electrodes,^67^ whereas uric acid generally exhibits a weak oxidation signal around +0.3 V vs Ag|AgCl|KCl_(sat.)_, becoming clearly detectable only at concentrations above 50 μmol L^–1^ when using a glassy carbon electrode under comparable experimental conditions.^68^ These characteristics account for the absence of discernible oxidation peaks for these species in the voltammograms presented in Figure S10C,D. In the presence of uric acid, only a slight shift in the oxidation potential of 5-HT was observed, from +0.32 V to +0.28 V vs Ag|AgCl|KCl_(sat.)_, without significant attenuation of the analytical signal. Regarding epinephrine (Figure S10E) and dopamine (Figure S10F), both compounds exhibited a well-defined and intense oxidation process at approximately +0.13 V vs Ag|AgCl|KCl_(sat.)_. Importantly, these oxidation peaks were clearly separated from the 5-HT signal and did not compromise either the peak position or the current intensity associated with the analyte. Taken together, these results, along with the bar diagram demonstrating that the relative variation in the 5-HT electrochemical response in the presence of each potential interferent remained below 10% (see Figure S10G), confirm that the proposed sensor maintains its analytical performance in the presence of coexisting species. Therefore, the device demonstrates satisfactory selectivity and is well-suited for reliable determination of 5-HT in complex biological matrices.

### 3.5 Application of the Proposed Method to Biological Matrices

After careful optimization of the proposed analytical method and a thorough evaluation of the figures of merit, the improved coumarin-benzothiazole-based sensor was applied to the proof-of-concept determination of 5-HT in complex biological matrices, specifically human serum and cerebrospinal fluid samples. Under the initial experimental conditions, no detectable electrochemical signal attributable to endogenous 5-HT was observed in either matrix. Consequently, the samples were spiked at two concentration levels (0.5 and 1.0 μmol L^–1^), and standard-addition calibration curves were constructed directly in the presence of the sample matrix to account for possible matrix effects (Figures 9 and S11). As summarized in Table 2, recovery values close to 100% were obtained for both biological matrices at both concentration levels investigated, with satisfactory precision. Overall, the analytical performance observed in complex samples highlights the strong potential of the proposed coumarin-benzothiazole-based sensor for electroanalytical applications involving biological fluids, particularly for sensitive and selective 5-HT determination.

**Figure 9:**
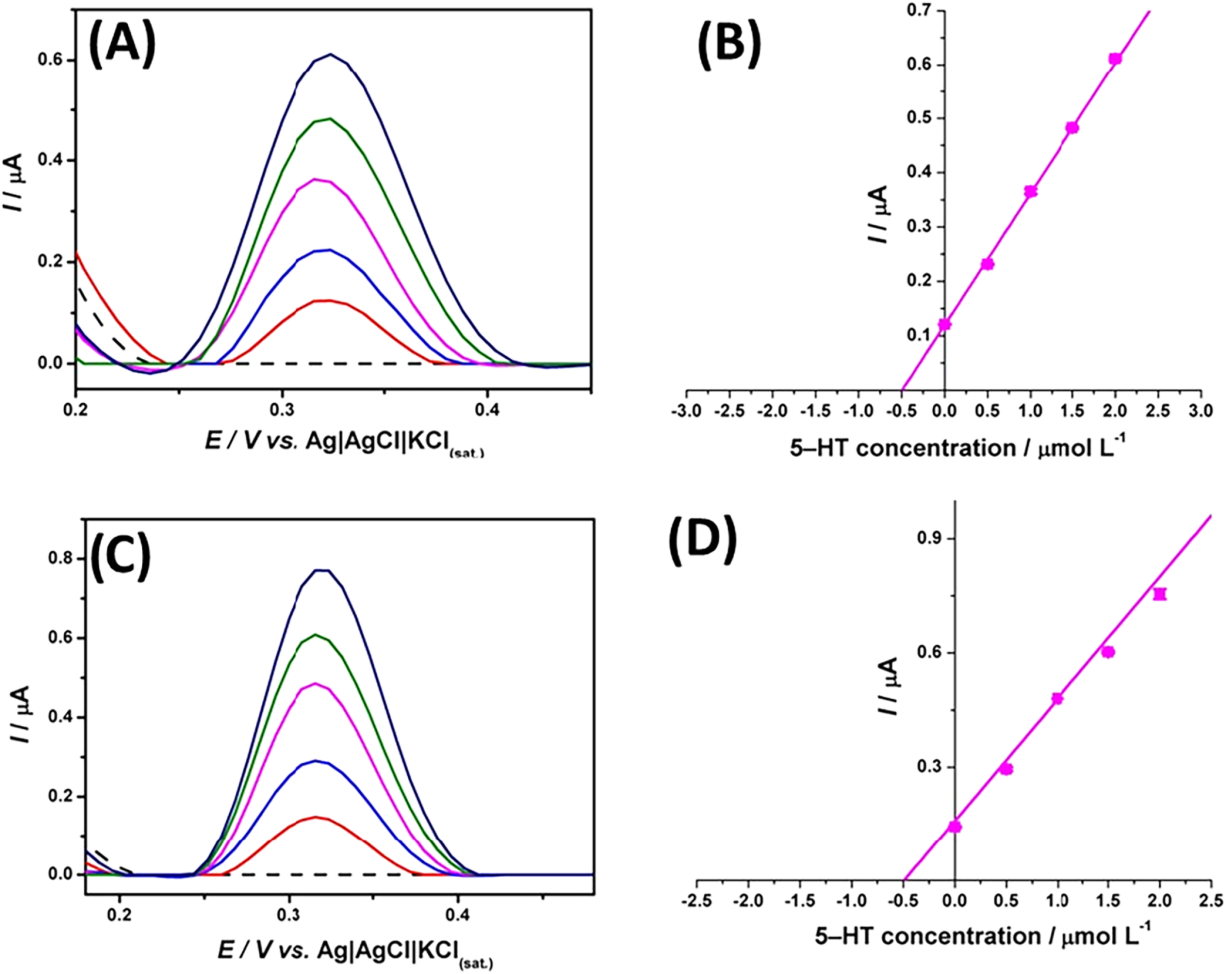
DPV responses (*n* = 3) were obtained for human serum (A) and cerebrospinal fluid (C) using 3D-printed G/coumarin-benzothiazole/PLA electrodes. The respective calibration plots by the standard addition method are represented by (B and D). The voltammograms show the nonspiked sample (black lines), and spiked sample at 0.5 μmol L^–1^ 5–HT (red line), followed by the four standard additions (blue, magenta, green, and blue lines). DPV conditions: modulation amplitude of 70 mV, scan rate of 30 mV s^–1^, and step potential of 8 mV. Background electrolyte: 0.12 mol L^–1^ phosphate buffer (pH 7.0).

**Table 2:** 5-HT Contents Determined by the Proposed Method

Ssample	Spiked/μmol L^–1^	Found/ μmol L^–1^	Recovery/%
Human serum	0.50	0.51 ± 0.15	102.4 ± 1.3
	1.00	0.98 ± 0.10	98.0 ± 1.8
Cerebrospinal fluid	0.50	0.47 ± 0.11	94.0 ± 2.2
	1.00	1.01 ± 0.33	101.2 ± 2.3

The analytical performance of the developed sensor was systematically benchmarked against electrochemical platforms previously reported in the literature, considering parameters such as sensor architecture, electroanalytical technique, linear dynamic range, LODs, and the nature of the evaluated matrices, as summarized in Table 3. The proposed 3D-G/Coumarin–benzothiazole/PLA (30:5:65 wt. %) sensor operated within a linear working range of 0.1–6.0 μM, well suited to the typical 5-HT concentrations found in clinically relevant biological matrices. Notably, the LOD achieved (0.07 μM) is within the same order of magnitude and, in some cases, superior to those reported for more sophisticated and costly electrode architectures, including systems based on noble metals,^61,69^ carbon nanotube composites,^60,70^ and nanostructured hybrid materials.^71^ In terms of real-sample applicability, the sensor demonstrated reliable performance in complex biological matrices, such as human serum and cerebrospinal fluid, highlighting its suitability for clinically relevant 5-HT monitoring. Moreover, the proposed platform offers clear advantages in terms of simple fabrication, the use of sustainable and low-cost materials, and robust analytical performance, with satisfactory accuracy, precision, and selectivity, thus positioning it as a promising 3D-printed carbon-based sensing platform for routine 5-HT determination.

**Table 3:** An Overview of the Analytical Performance of the Proposed Sensor toward 5-HT Detection, Compared with Electrochemical Systems Previously Described in the Literature

Sensor	Method	Linear range (μmol L^–1^)	LOD (μmol L^–1^)	Samples	Refs
**DecA:TBAB** (5 mg)-CNH/GCE	SWV	0.50–32.71	0.09	Synthetic urine and bovine serum	^64^
**Pt/MWCNT/PPy/AgNPs**	DPV	0.5–5.0	0.15	Plasmatic serum	^71^
**MWCNT-PANI/GCE**	DPV	0.1–12.0	0.03	Synthetic urine	^70^
**P-WO3 NS/GCE**	LSV	0.01–100.0	0.60	Human urine and blood serum samples	^69^
**NDs-AuNPs-Gr-CSN/GCE**	DPV	0.3–3.0	0.10	Synthetic urine	^61^
**3D-G/coumarin-benzothiazole/PLA** **(30:5:65** **wt.** **%)**	DPV	0.1–6.0	0.07	Human Serum and Cerebrospinal fluid	**This work**

**DecA:TBAB­(5 mg)-CNH/GCE:** *Electrode modified with HDES and carbon nanohorns;* **Pt/MWCNT/PPy/AgNPs:** *platinum electrode modified with carbon nanotubes/polypyrrole/silver nanoparticles nanohybrid* **; MWCNT-PANI:** *multiwalled carbon nanotube-poly (aniline);* **P-WO3 NS** *: phosphorus-doped tungsten trioxide nanosheet* **; NDs-AuNPs- Gr-CSN:** *a film based on incorporation of nanodiamonds, gold nanoparticles, and graphite anchored in casein;* **SWV:** *square wave voltammetry;* **DPV:** *differential pulse voltammetry;* **LSV**: *Linear Sweep Voltammetry*.

## 4 Conclusion

In this study, a sustainable and multifunctional 3D-printed composite platform based on coumarin–benzothiazole, PLA, and graphite was developed for the rapid, scalable, and cost-effective fabrication of high-performance electrochemical sensors. All components were thoroughly characterized using spectroscopic, morphological, elemental, and electrochemical techniques, confirming the successful incorporation and homogeneous dispersion of graphite and coumarin–benzothiazole in the PLA matrix. The presence of the organic modifier played a key role in modulating the electrode interfacial properties, promoting enhanced electrochemical activity, faster charge-transfer kinetics, and higher sensitivity toward 5-HT detection. The proposed 3D-G/coumarin-benzothiazole/PLA (30:5:65 wt. %) sensor exhibited excellent analytical performance, with high sensitivity, selectivity, and operational stability, even in complex biological matrices. Reliable quantification of 5-HT in human serum and cerebrospinal fluid demonstrates the platform’s practical applicability for clinically relevant analyses. Overall, the strategy of integrating a functional organic molecule with a conductive carbon phase into a thermoplastic matrix through a simple solution-processing and drying approach represents a scalable pathway for producing customizable electrochemical sensing platforms compatible with additive manufacturing technologies. This work highlights the potential of organic-modified 3D-printable composites as a new generation of low-cost, high-performance electrochemical devices for biomedical and analytical monitoring.

## Supplementary Material



## Notes

X-ray diffraction (XRD) patterns of the G/PLA and G/coumarin-benzothiazole/PLA composites; voltammetric studies for electrode characterization; electrochemical analysis of actual samples; and supplementary tables (PDF)

## References

[1] Jayakrishna M., Vijay M., Khan B. (2023). An Overview of Extensive Analysis
of 3D Printing Applications in the Manufacturing Sector. J. Eng..

[2] Shahrubudin N., Lee T. C., Ramlan R. (2019). An Overview on 3D Printing Technology:
Technological, Materials, and Applications. Procedia Manuf..

[3] Cardoso R. M., Kalinke C., Rocha R. G., dos Santos P. L., Rocha D. P., Oliveira P. R., Janegitz B. C., Bonacin J. A., Richter E. M., Munoz R. A. A. (2020). Additive-Manufactured (3D-Printed)
Electrochemical Sensors: A Critical Review. Anal. Chim. Acta.

[4] Stefano J. S., Kalinke C., da Rocha R. G., Rocha D. P., da Silva V. A. O. P., Bonacin J. A., Angnes L., Richter E. M., Janegitz B. C., Muñoz R. A. A. (2022). Electrochemical (Bio)­Sensors Enabled
by Fused Deposition
Modeling-Based 3D Printing: A Guide to Selecting Designs, Printing
Parameters, and Post-Treatment Protocols. Anal.
Chem..

[5] Silva A. L., da Silva Salvador G.
M., Castro S. V. F., Carvalho N. M. F., Munoz R. A. A. (2021). A 3D
Printer Guide for the Development and Application of Electrochemical
Cells and Devices. Front. Chem..

[6] Rocha D. P., Rocha R. G., Castro S. V. F., Trindade M. A. G., Munoz R. A. A., Richter E. M., Angnes L. (2021). Posttreatment of 3D-printed Surfaces
for Electrochemical Applications: A Critical Review on Proposed Protocols. Electrochem. Sci. Adv..

[7] Crapnell R. D., Kalinke C., Silva L. R. G., Stefano J. S., Williams R. J., Munoz R. A. A., Bonacin J. A., Janegitz B. C., Banks C. E. (2023). Additive
Manufacturing Electrochemistry: An Overview of Producing Bespoke Conductive
Additive Manufacturing Filaments. Mater. Today.

[8] Kalinke C., Neumsteir N. V., de Oliveira Aparecido G., de Barros
Ferraz T. V., dos Santos P. L., Janegitz B. C., Bonacin J. A. (2020). Comparison
of Activation Processes for 3D Printed PLA-Graphene Electrodes: Electrochemical
Properties and Application for Sensing of Dopamine. Analyst.

[9] Silva J. P. C., Rocha R. G., Siqueira G. P., Nascimento C. F., Santana M. H. P., Nossol E., Richter E. M., da Silva I. S., Muñoz R. A. A. (2025). Bio-Based Plasticizer Babassu Oil
for Custom-Made Conductive
Additive-Manufacturing Filaments: Towards 3D-Printed Electrodes Applied
to Cocaine Detection. Microchimica Acta.

[10] Kokkinos C. (2025). 3D-Printed
Thermoplastic Sensors for Electrochemical Biosensing. Curr. Opin. Electrochem..

[11] Stefanachi A., Leonetti F., Pisani L., Catto M., Carotti A. (2018). Coumarin:
A Natural, Privileged and Versatile Scaffold for Bioactive Compounds. Molecules..

[12] Abbas W. R., Kadhim M. A. (2025). Design and Synthesis
of New Coumarin-Based Fluorescent
Chemosensors for the Dual Detection of Hg2+ and Cu2+ in Aqueous and
Biological Samples. RSC Adv..

[13] Das S., Indurthi H. K., Saha P., Sharma D. K. (2024). Coumarin-Based Fluorescent
Probes for the Detection of Ions, Biomolecules and Biochemical Species
Responsible for Diseases. Dye Pigm..

[14] B M., Bodke Y. D., Mounesh, Bhat S. A. (2024). Coumarin-Based Composite
Material
for the Latent Fingerprint Visualization and Electrochemical Sensing
of Hydrogen Peroxide. RSC Sustainability.

[15] Moscoso R., Barrientos C., Abarca S., Squella J. A. (2023). Electrochemical
Characterization of Nitrocoumarin-Modified Nanostructured Electrode
Platforms: New Precursors for the Electrocatalysis of NADH. Electrochim. Acta.

[16] Guruprasadagowda Y. K., Harish M. N. K., Tripathy D., Sampath S. (2022). Tetrakis Coumarin as
Efficient Electrode Material for Rechargeable Lithium Ion Battery. J. Electroanal. Chem..

[17] Feng D., Zhang A., Yang Y., Yang P. (2020). Coumarin-Containing
Hybrids and Their Antibacterial Activities. Arch. Pharm..

[18] Cheke R. S., Patel H. M., Patil V. M., Ansari I. A., Ambhore J. P., Shinde S. D., Kadri A., Snoussi M., Adnan M., Kharkar P. S., Pasupuleti V. R., Deshmukh P. K. (2022). Molecular Insights
into Coumarin Analogues as Antimicrobial Agents: Recent Developments
in Drug Discovery. Antibiotics..

[19] Prusty J. S., Kumar A. (2020). Coumarins: Antifungal Effectiveness and Future Therapeutic Scope. Mol. Diversity.

[20] Bhattarai N., Kumbhar A. A., Pokharel Y. R., Yadav P. N. (2021). Anticancer Potential
of Coumarin and Its Derivatives. Mini-Rev. Med.
Chem..

[21] Kostova I., Bhatia S., Grigorov P., Balkansky S., Parmar V. S., Prasad A. K., Saso L. (2011). Coumarins
as Antioxidants. Curr. Med. Chem..

[22] Kasperkiewicz K., Ponczek M. B., Owczarek J., Guga P., Budzisz E. (2020). Antagonists
of Vitamin K-Popular Coumarin Drugs and New Synthetic and Natural
Coumarin Derivatives. Molecules.

[23] Lin T. H., Chang K. H., Chiu Y. J., Weng Z. K., Sun Y. C., Lin W., Lee-Chen G. J., Chen C. M. (2022). Neuroprotective Action of Coumarin
Derivatives through Activation of TRKB-CREB-BDNF Pathway and Reduction
of Caspase Activity in Neuronal Cells Expressing Pro-Aggregated Tau
Protein. Int. J. Mol. Sci..

[24] Crapnell R. D., Bernalte E., Banks C. E. (2026). Sustainable
Advancements in Fused
Filament Fabrication/Fused Deposition Modelling Additive Manufacturing
for Electroanalysis. Green Chem..

[25] Caldas N. M., de Faria L. V., Batista A. G., Alves A. O., Silva S. C., Peixoto D. A., Nossol E., Rocha D. P., Semaan F. S., Pacheco W. F., Dornellas R. M. (2024). Graphite
and Silver Nanoparticles-Loaded
Polylactic Acid Matrix: A Pioneering Tailor-Lab-Made Filament for
Manufacturing Eco-Friendly and Robust Electrochemical Sensors towards
Pyridoxine Detection. Electrochim. Acta.

[26] Stefano J. S., e Silva L. R. G., Rocha R. G., Brazaca L. C., Richter E. M., Muñoz R. A. A., Janegitz B. C. (2022). New Conductive Filament Ready-to-Use
for 3D-Printing Electrochemical (Bio)­Sensors: Towards the Detection
of SARS-CoV-2. Anal. Chim. Acta.

[27] Brum R. R. D., de Faria L. V., Caldas N. M., Pereira R. P., Peixoto D. A., Silva S. C., Nossol E., Semaan F. S., Pacheco W. F., Rocha D. P., Dornellas R. M. (2025). 3D-Printed
Electrochemical Sensor
Based on Graphite-Alumina Composites: A Sensitive and Reusable Platform
for Self-Sampling and Detection of 2,4,6-Trinitrotoluene Residues
in Environmental and Forensic Applications. Talanta Open.

[28] de
Faria L. V., Villafuerte L. M., do Nascimento S. F. L., de Sá I. C., Peixoto D. A., Ribeiro R. S., de A., Nossol E., Lima T., de M., Semaan F. S., Pacheco W. F., Dornellas R. M. (2024). 3D-Printed Electrodes Using Graphite/Carbon
Nitride/Polylactic Acid Composite Material: A Greener Platform for
Detection of Amaranth Dye in Food Samples. Food
Chem..

[29] de
Toledo R. A., Santos M. C., Cavalheiro E. T. G., Mazo L. H. (2005). Determination of Dopamine in Synthetic Cerebrospinal
Fluid by SWV with a Graphite–Polyurethane Composite Electrode. Anal. Bioanal. Chem..

[30] Miranda F. S., Ronconi C., Sousa M., De Queiros SIlveira G., Vargas M. (2013). 6-Aminocoumarin-Naphthoquinone Conjugates:
Design,
Synthesis, Photophysical and Electrochemical Properties and DFT Calculations. J. Braz. Chem. Soc..

[31] Lima J. G. M., dos Santos J. R. N., Abegão L. M. G., Cocca L. H. Z., de
Almeida L. R., Napolitano H. B., Ribeiro L., Ramos L. M. (2025). Synthesis,
Characterization, and
Dual Functional Properties of Coumarin-Based Hybrids for Biological
and Optical Applications. ACS Omega.

[32] Abdallah A. E. M., Abdel-Latif S. A., Elgemeie G. H. (2023). Novel Fluorescent
Benzothiazolyl-Coumarin Hybrids as Anti-SARS-COVID-2 Agents Supported
by Molecular Docking Studies: Design, Synthesis, X-Ray Crystal Structures,
DFT, and TD-DFT/PCM Calculations. ACS Omega.

[33] Das N., Hossain A., Chand B., Ghanta S., Sieroń L., Maniukiewicz W., Misra T. K. (2026). Synthesis, Spectral Characterization,
Structures, and Computation of 1-Methyl-/1,3-Dimethyl-5-(p-Nitro-Phenylazo)-6-Aminouracils
and Silver­(I) Complexes. J. Mol. Struct..

[34] Ling J., Rong M. Z., Zhang M. Q. (2011). Coumarin
Imparts Repeated Photochemical
Remendability to Polyurethane. J. Mater. Chem..

[35] Kaczor D., Fiedurek K., Bajer K., Raszkowska-Kaczor A., Domek G., Macko M., Madajski P., Szroeder P. (2021). Impact of
the Graphite Fillers on the Thermal Processing of Graphite/Poly­(Lactic
Acid) Composites. Materials.

[36] González-López M., González-López J. A., Reyes-Morales Q. L., Pereyra I., Mayen J. (2024). Modifying the Manufacturing Process
of High-Graphite Content Polylactic Acid Filament for Advanced Energy
and Sensing Applications in 3D Printing. Polymer.

[37] dos
Santos A. L., de Souza F. C. R., da Costa J. C. M., Gonçalves D. A., Passos R. R., Pocrifka L. A. (2024). Development and Characterization
of 3D-Printed PLA/Exfoliated Graphite Composites for Enhanced Electrochemical
Performance in Energy Storage Applications. Polymers.

[38] Miranda F. S., Ronconi C. M., Sousa M. O. B., Silveira G. Q., Vargas M. D. (2013). 6-Aminocoumarin-Naphthoquinone
Conjugates: Design, Synthesis, Photophysical and Electrochemical Properties
and DFT Calculations. J. Braz. Chem. Soc..

[39] Yang Z., Peng H., Wang W., Liu T. (2010). Crystallization Behavior
of Poly­(ε-Caprolactone)/Layered Double Hydroxide Nanocomposites. J. Appl. Polym. Sci..

[40] Yasmin A., Luo J. J., Daniel I. M. (2006). Processing of Expanded Graphite Reinforced
Polymer Nanocomposites. Compos. Sci. Technol..

[41] Mohammadi
Zerankeshi M., Sayedain S. S., Tavangarifard M., Alizadeh R. (2022). Developing a Novel Technique for the Fabrication of
PLA-Graphite Composite Filaments Using FDM 3D Printing Process. Ceram. Int..

[42] Li Z. Q., Lu C. J., Xia Z. P., Zhou Y., Luo Z. (2007). X-Ray Diffraction
Patterns of Graphite and Turbostratic Carbon. Carbon N. Y..

[43] Roscher S., Hoffmann R., Ambacher O. (2019). Determination of the
Graphene-Graphite
Ratio of Graphene Powder by Raman 2D Band Symmetry Analysis. Anal. Methods.

[44] Bessa I. A. A., Cruz J. V. R., de Sousa M. O. B., Marques F. D., Archanjo B. S., Rocco M. L. M., Nascimento V., Pinto L. F. R., dos
Santos T. C., Costa N. M., Ronconi C. M. (2025). Interfacing Reduced
Graphene Oxide with Cationic Pillar[5]­Arene for Doxorubicin Delivery:
A Platform for Glioblastoma Treatment. ACS Appl.
Nano Mater..

[45] Qiu T., Yang J. G., Bai X. J., Wang Y. L. (2019). The Preparation
of Synthetic Graphite Materials with Hierarchical Pores from Lignite
by One-Step Impregnation and Their Characterization as Dye Absorbents. RSC Adv..

[46] Zhao W., Qiao Y., Jia H., Zhang Y., Zhang E., Jian X., Liu C. (2025). 2D GO and
3D NH2-UiO-66 Assembled
on Carbon Fiber Surface by Electrodeposition Method for Enhancing
the Interfacial Performance of CF/PBPESK Composite. Chem. Eng. J..

[47] Layek R. K., Nandi A. K. (2013). A Review on Synthesis and Properties
of Polymer Functionalized
Graphene. Polymer.

[48] Bokobza L., Bruneel J.-L., Couzi M. (2015). Raman Spectra
of Carbon-Based Materials
(from Graphite to Carbon Black) and of Some Silicone Composites. C.

[49] Madito M. J. (2025). Revisiting
the Raman Disorder Band in Graphene-Based Materials: A Critical Review. Vib. Spectrosc..

[50] Uesugi Y., Mizuno M., Shimojima A., Takahashi H. (1997). Transient
Resonance Raman and Ab Initio MO Calculation Studies of the Structures
and Vibrational Assignments of the T1 State and the Anion Radical
of Coumarin and Its Isotopically Substituted Analogues. J. Phys. Chem. A.

[51] Bozzini B., D’Urzo L. (2009). An In-Situ
SERS Study of the Interfacial Behaviour
of Coumarin during the Electrodeposition of Cobalt. Int. J. Electrochem. Sci..

[52] Elnobi S., Abdou M. M., Abuelwafa A. A. (2025). Eco-Friendly
One-Pot Synthesis, Structural,
and Physical Properties of Coumarin 6. Opt.
Laser Technol..

[53] Pérez E. M., Martín N. (2015). π-π
Interactions in Carbon Nanostructures. Chem.
Soc. Rev..

[54] Krishnan R. G., Saraswathyamma B. (2021). Disposable Electrochemical Sensor for Coumarin Induced
Milk Toxicity in Raw Milk Samples. Measurement.

[55] Dempsey E., O’Sullivan C., Smyth M. R., Egan D., O’Kennedy R., Wang J. (1993). Differential Pulse Voltammetric Determination of 7-Hydroxycoumarin
in Human Urine. J. Pharm. Biomed. Anal..

[56] Lazanas A. C., Prodromidis M. I. (2023). Electrochemical Impedance Spectroscopy–A Tutorial. ACS Meas. Sci. Au.

[57] González-Meza O. A., Larios-Durán E. R., Gutiérrez-Becerra A., Casillas N., Escalante J. I., Bárcena-Soto M. (2019). Development
of a Randles-Ševčík-like Equation to Predict
the Peak Current of Cyclic Voltammetry for Solid Metal Hexacyanoferrates. J. Solid State Electrochem..

[58] Oldham K. B. (1979). Analytical
Expressions for the Reversible Randles-Sevcik Function. J. Electroanal. Chem. Interfacial Electrochem..

[59] Laviron E. (1979). General Expression
of the Linear Potential Sweep Voltammogram in the Case of Diffusionless
Electrochemical Systems. J. Electroanal. Chem.
Interfacial Electrochem..

[60] de
Lima Augusto K. K., Gomes-Junior P. C., Longatto G. P., Piccin E., Cavalheiro É. T. G., Bernalte E., Banks C. E., Fatibello-Filho O. (2025). Electrochemical Sensor Based on Carbon Nanohorns and
Hydrophobic Deep Eutectic Solvent for the Determination of Serotonin
in Biological Samples. Electrochim. Acta.

[61] Ramos M. M. V., Carvalho J. H. S., de
Oliveira P. R., Janegitz B. C. (2020). Determination of Serotonin by Using
a Thin Film Containing
Graphite, Nanodiamonds and Gold Nanoparticles Anchored in Casein. Measurement.

[62] Silva V. A. O. P., Fernandes-Junior W. S., Rocha D. P., Stefano J. S., Munoz R. A. A., Bonacin J. A., Janegitz B. C. (2020). 3D-Printed Reduced
Graphene Oxide/Polylactic Acid Electrodes: A New Prototyped Platform
for Sensing and Biosensing Applications. Biosens.
Bioelectron..

[63] Ahmed Y. M., El-Zanaty M. R., Galal A., Atta N. F. (2025). Voltammetry Sensor
Based on Poly­(L-Methionine)/Reduced Graphene Oxide/β-Cyclodextrin
for the Simultaneous Determination of Caffeine, Dopamine and Serotonin. Food Chem..

[64] de
Lima Augusto K. K., Gomes-Junior P. C., Longatto G. P., Piccin E., Cavalheiro É. T. G., Bernalte E., Banks C. E., Fatibello-Filho O. (2025). Electrochemical Sensor Based on Carbon Nanohorns and
Hydrophobic Deep Eutectic Solvent for the Determination of Serotonin
in Biological Samples. Electrochim. Acta.

[65] Long G. L., Winefordner J. D. (1983). Limit of
Detection. A Closer Look at the IUPAC Definition. Anal. Chem..

[66] Caldas N. M., de Faria L. V., Batista A. G., Alves A. O., de Souza C. C., Borges P. H. S., Nossol E., Matos R. C., Rocha D. P., Semaan F. S., Dornellas R. M. (2025). Lab-Created Conductive Filament Based
on Nickel and Graphite Particles: An Attractive Material for the Additive
Manufacture of Enhanced Electrochemical Sensors for Non-Enzymatic
and Selective Glucose Sensing. Talanta.

[67] Caldas N. M., Batista A. G., de Carvalho G. S. G., D’ Amato D. L., Ronconi C. M., da Silva F., de C., de Faria L. V., Rocha D. P., Dornellas R. M. (2026). A New Carbon–Kaolinite
Composite
Conductive Filament for 3D-Printed Electrochemical Tyrosine Sensors. Electrochim. Acta.

[68] Nakayama M., Wakamiya M., Jin J. (2026). Determination
of Uric Acid in Human
Urine by Differential Normal Pulse Voltammetry With a Bare Glassy
Carbon Electrode. Electroanalysis.

[69] Babulal S. M., Wu H.-F., Chien
Wu C. (2023). Two-Dimensional Phosphorus-Doped
Tungsten Trioxide Nanosheets for Electrochemical Detection of Serotonin
in Biological Fluids. ACS Appl. Nano Mater..

[70] Koluaçık E., Karabiberoğlu ŞU., Dursun Z. (2018). Electrochemical Determination
of Serotonin Using Pre-Treated Multi-Walled Carbon Nanotube-Polyaniline
Composite Electrode. Electroanalysis.

[71] Cesarino I., Galesco H. V., Machado S. A. S. (2014). Determination of Serotonin on Platinum
Electrode Modified with Carbon Nanotubes/Polypyrrole/Silver Nanoparticles
Nanohybrid. Mater. Sci. Eng.: C.

