# Supplementary material for: Homemade Filament Incorporating an Organic Modifier (Coumarin-Benzothiazole) for the Fabrication of High-Performance Voltammetric Sensors for Serotonin Determination
Source: ACS Omega. 2026 Jul 23;11(30):45536–48. doi: 10.1021/acsomega.6c06229 (PMC13448931; doi:10.1021/acsomega.6c06229)
Supplement: Supplementary file 1 [file ao6c06229_si_001.pdf]

Homemade Filament Incorporating an Organic Modifier (Coumarin-benzothiazole) for the Fabrication of High-Performance Voltammetric Sensors for Serotonin Determination

Natália M. Caldas,[1,#] Raíssa G. Gouvea,[2,#] Yuri P. V. de Carvalho,[2] Amanda G. Batista,[1] Mikaelly O. B. de Sousa,[3] Celia M. Ronconi,[3] Lucas V. de Faria,[4] Diego P. Rocha,[5] Luana da S. M. Forezi,[2,*] and Rafael M. Dornellas[1,*]

[1] Department of Analytical Chemistry, Institute of Chemistry, Fluminense Federal University, Niterói, 24020-141, RJ, Brazil.

[2] Department of Organic Chemistry, Institute of Chemistry, Fluminense Federal University, Niterói, 24020-141, RJ, Brazil.

[3] Department of Inorganic Chemistry, Institute of Chemistry, Federal Fluminense University, Niterói, 24020-141, RJ, Brazil.

[4] Institute of Chemistry, Federal University of Rio de Janeiro, Rio de Janeiro, 21941-909, RJ, Brazil.

[5] Department of Chemistry, Federal Institute of Paraná, Pitanga, 85201-106, PR, Brazil.

#contributed equally

*Corresponding authors: luanaforezi@id.uff.br; rafaeldornellas@id.uff.br

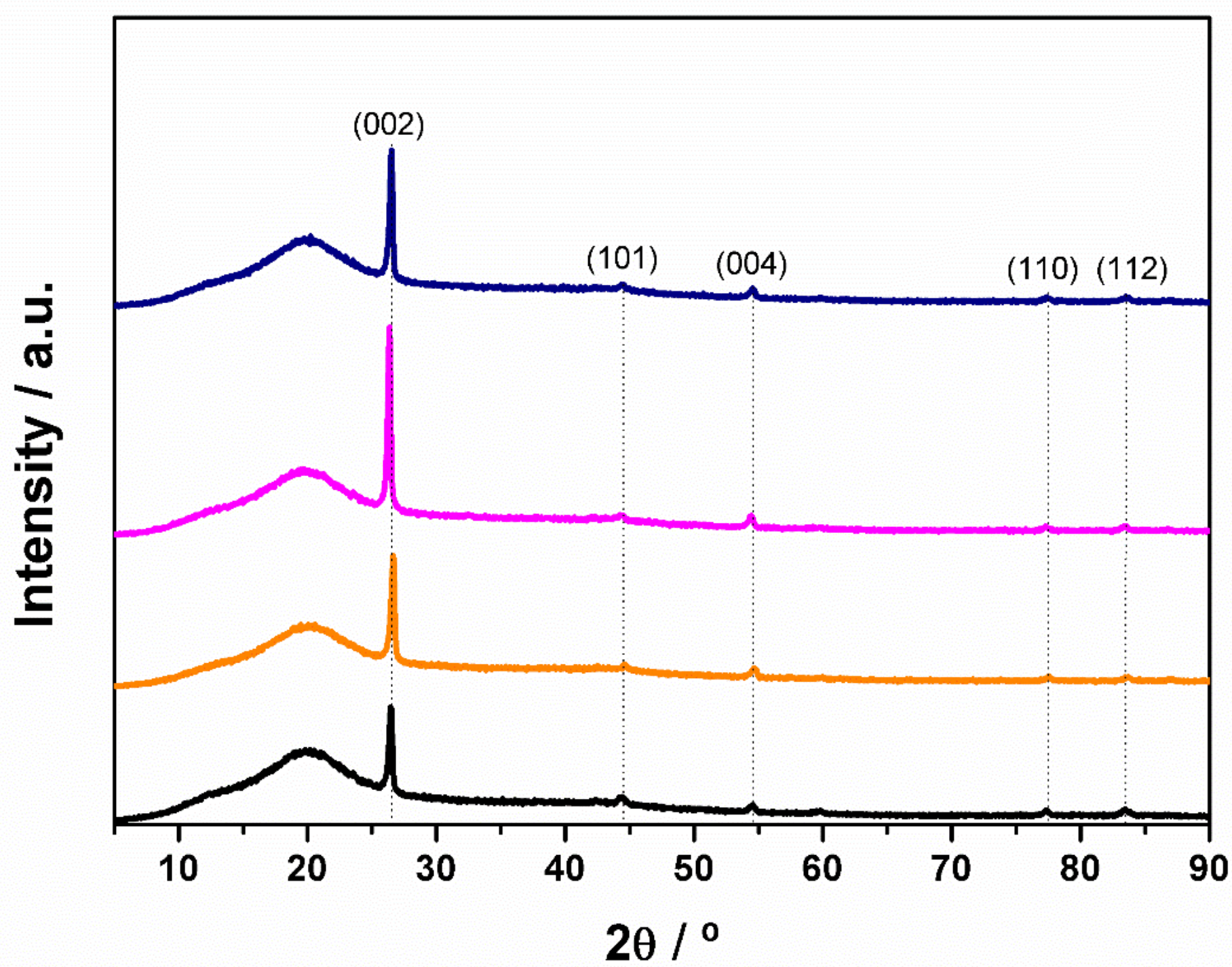

Figure S1. XRD patterns of the G/PLA (30:70 *wt.* %, black line), G/coumarin-benzothiazole/PLA (30:2.5:67.5 *wt.* %, orange line), G/coumarin-benzothiazole/PLA (30:5:65 *wt.* %, magenta line), and G/coumarin-benzothiazole/PLA (30:7.5:62.5 *wt.* %, dark blue line).

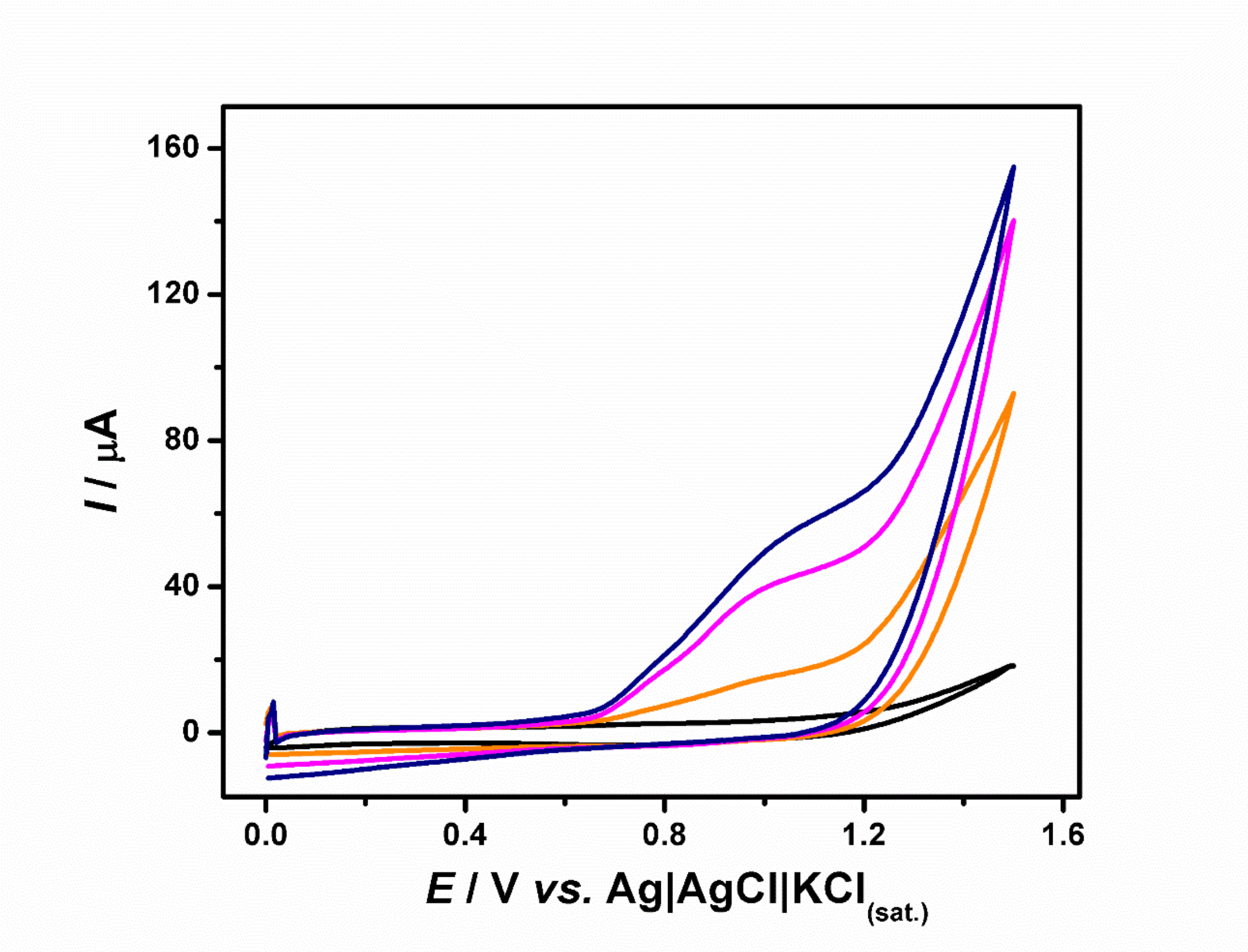

Figure S2. CV's electrochemical profiles obtained for the 3D-printed G/PLA (30:70 *wt.* %, black line), G/coumarin-benzothiazole/PLA (30:2.5:67.5 *wt.* %, orange line), G/coumarin-benzothiazole/PLA (30:5:65 *wt.* %, magenta line), and G/coumarin-benzothiazole/PLA (30:7.5:62.5 *wt.* %, dark blue line) electrodes using 0.1 mol L^{-1} KCl solution as the supporting electrolyte. CV conditions: scan rate: 50 mV s^{-1}; step potential: 5 mV.

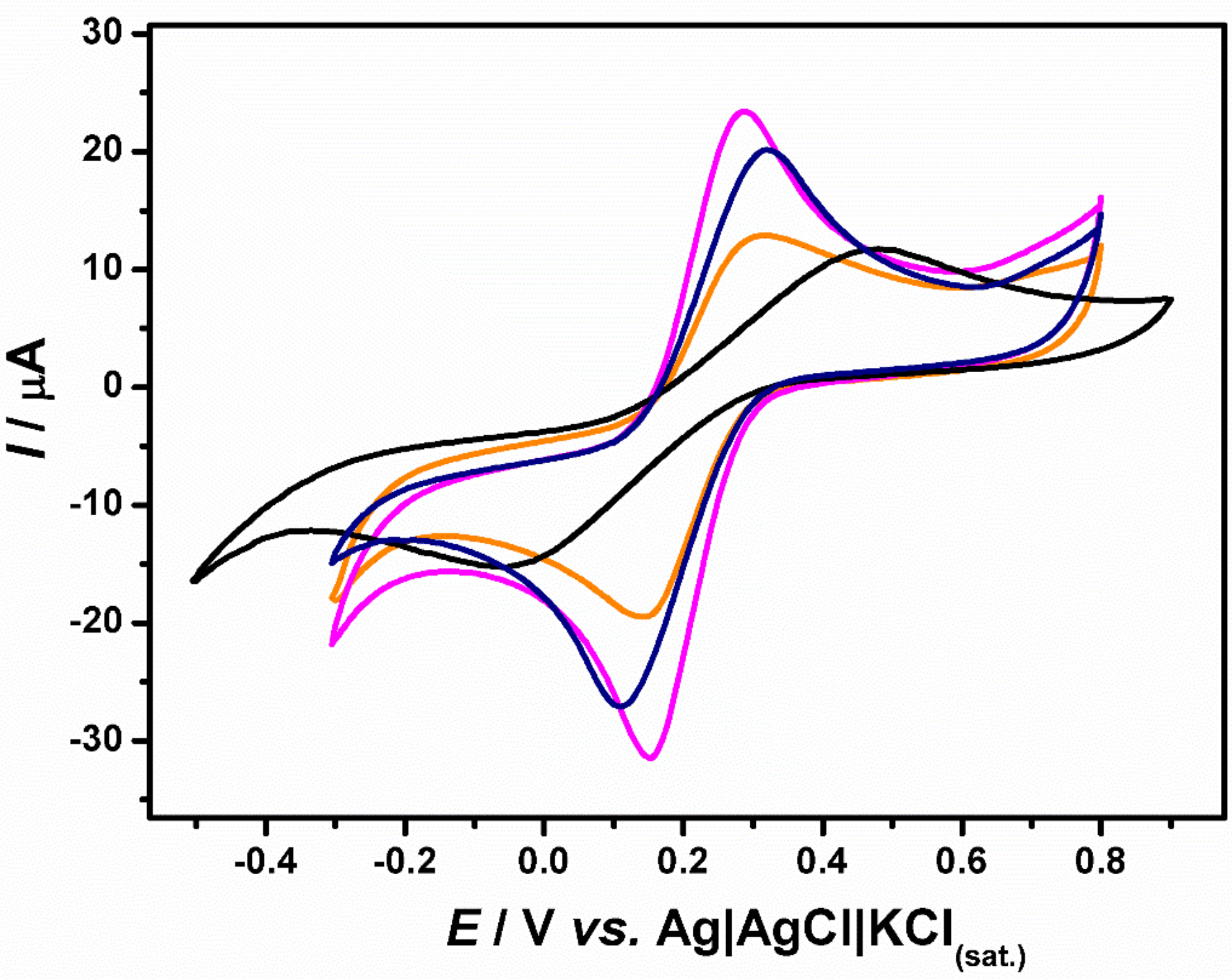

Figure S3. CVs recorded in the presence of 1mmol L^{-1} $[Fe(CN)_6]^{4-/3-}$ in 0.1 mol L^{-1} KCl (supporting electrolyte) medium using the G/PLA (30:70 *wt.* %, black line), G/coumarin-benzothiazole/PLA (30:2.5:67.5 *wt.* %, orange line), G/coumarin-benzothiazole/PLA (30:5:65 *wt.* %, magenta line), and G/coumarin-benzothiazole/PLA (30:7.5:62.5 *wt.* %, dark blue line). The dashed lines correspond to the respective blanks. Experimental conditions: scan rate of 50 mV s^{-1}; step potential of 5mV.

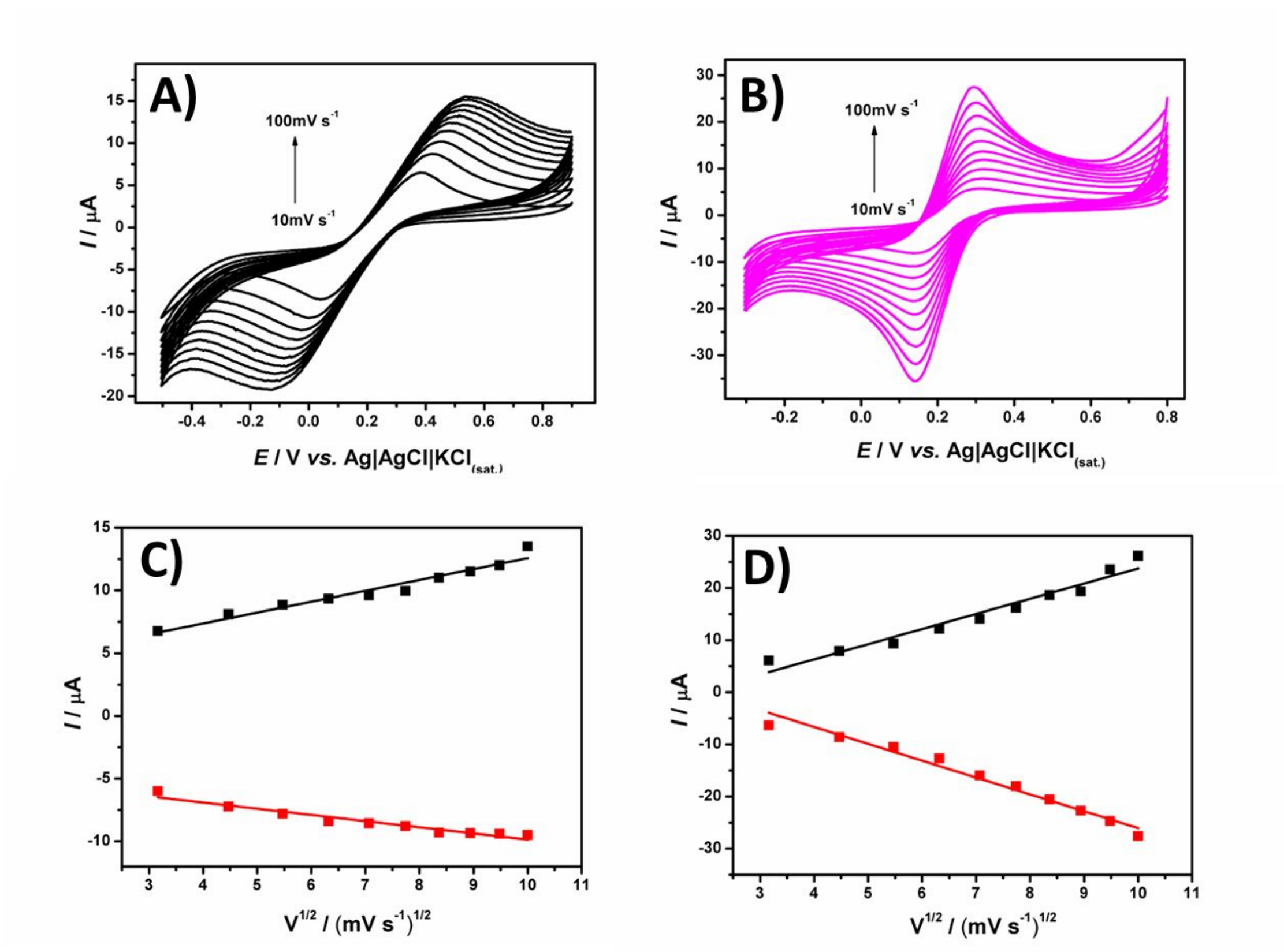

Figure S4. CVs responses recorded for 1 mmol L^{-1} $[Fe(CN)_6]^{4-/3-}$ in 0.1 mol L^{-1} KCl medium with scan rate ranging from 10 to 100 mV s^{-1} using (A) G/PLA (30:70 *wt.* %) and (B) G/coumarin-benzothiazole/PLA (30:5:65 *wt.* %) electrodes; (C) and (D) correspond to the respective linear adjustments between the peak current and the square root of the scan rate.

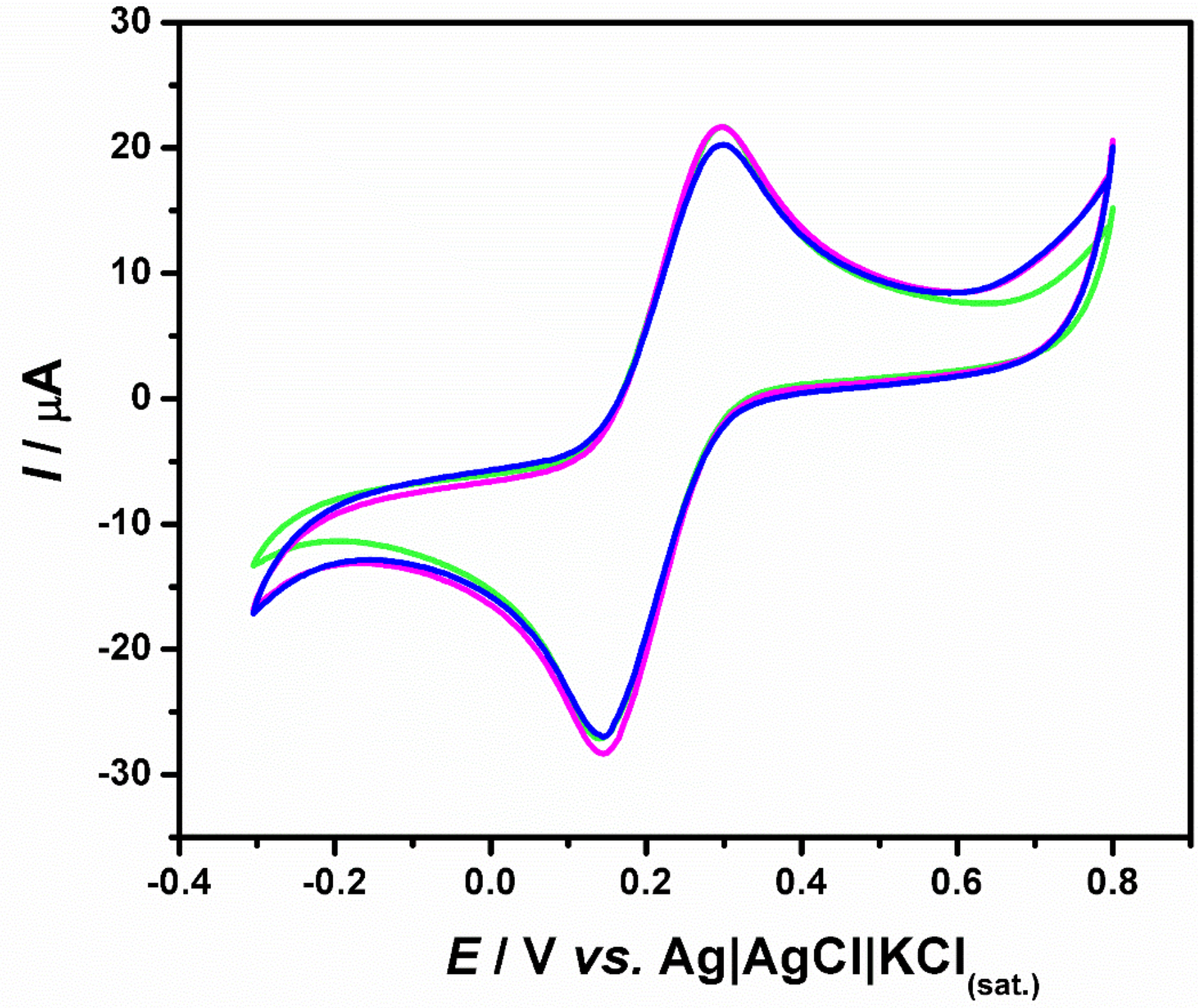

Figure S5. CVs recorded for 1 mmol L^{-1} $[Fe(CN)_6]^{4-/3-}$ in 0.1 mol L^{-1} KCl (background electrolyte) using three different (n = 3) 3D-printed G/coumarin-benzothiazole/PLA (30:5:65 *wt.* %) electrodes. CV conditions: scan rate of 50 mV s^{-1}; step potential of 5 mV.

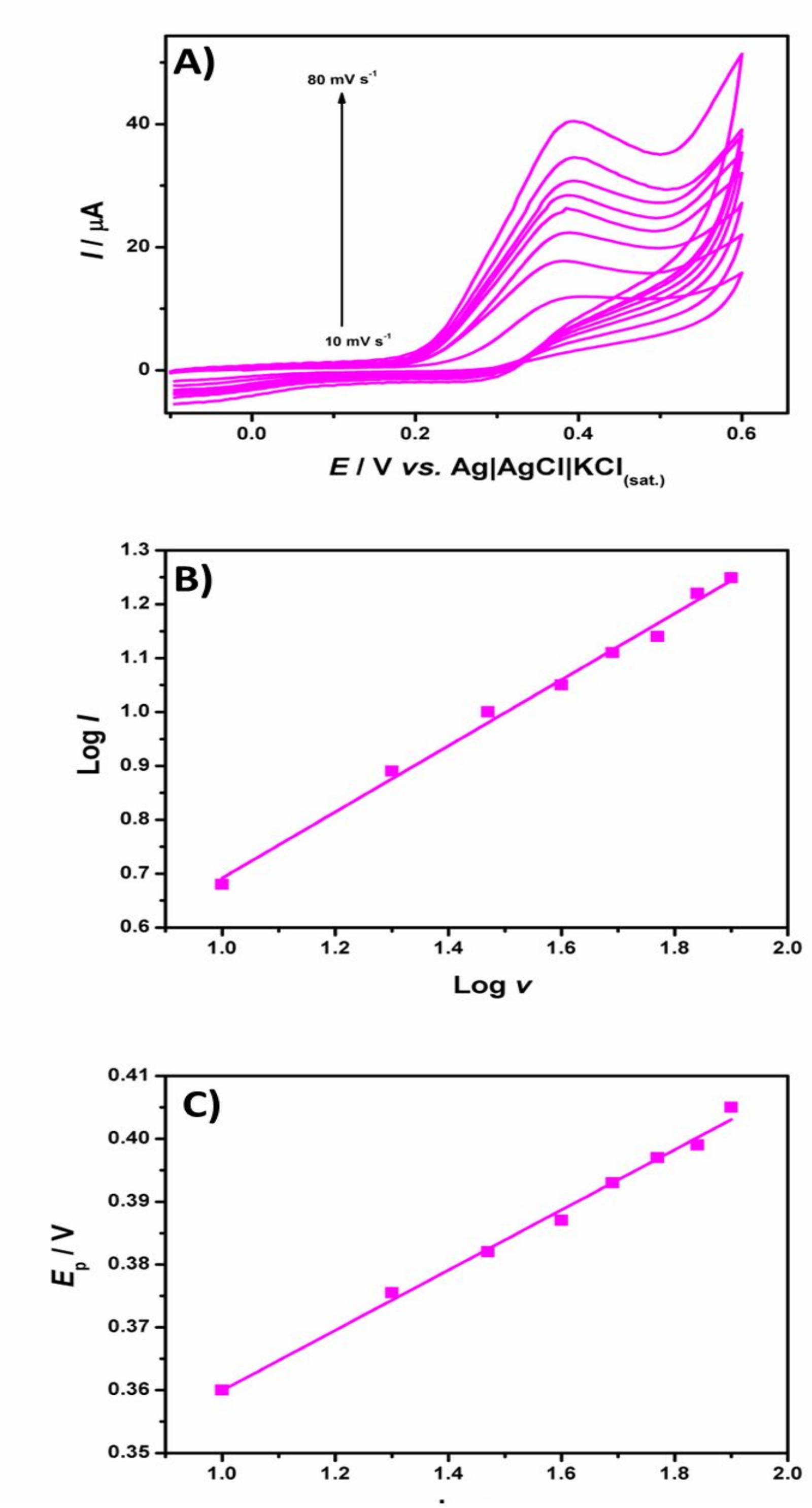

Figure S6. CV's measurements obtained for 0.5 mmol L^{-1} 5-HT with different scan rates (10 to 80 mV s^{-1}) utilizing a 3D-printed G/coumarin-benzothiazole/PLA (30:5:65 *wt.* %) electrode; (B) respective linear adjustment between Log *I* and Log *v* (C) E_p *vs.* Log *v*. Supporting electrolyte: 0.12 mol L^{-1} phosphate buffer (pH 7.0).

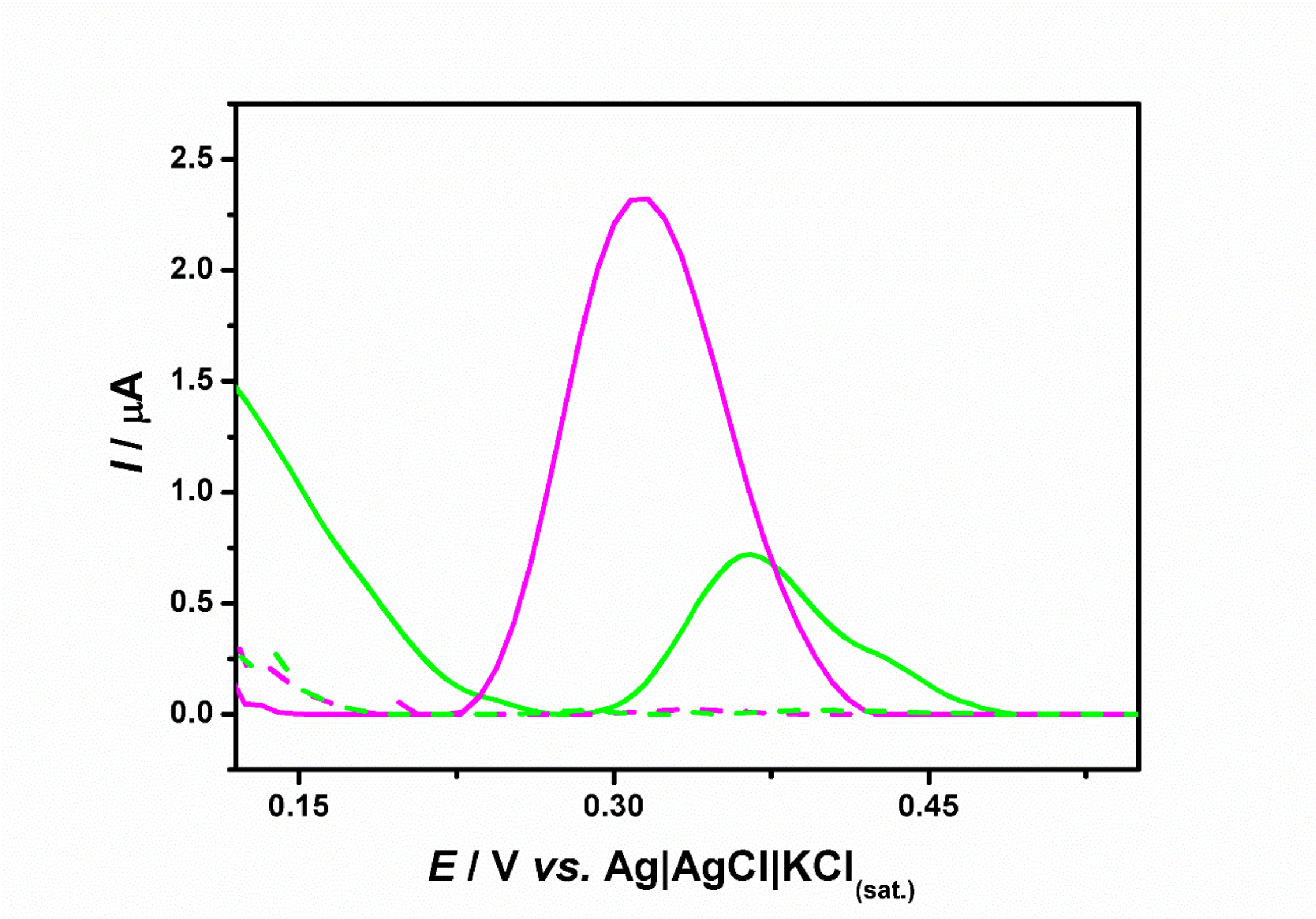

Figure S7. Electrochemical response of 4 μmol L^{-1} recorded using the G/coumarin-benzothiazole/PLA (30:5:65 *wt.* %) electrode by DPV (magenta line) and SWV (green line) techniques. DPV conditions: modulation amplitude of 70 mV, step potential of 8 mV, and scan rate of 30 mV s^{-1}; SWV conditions: modulation amplitude of 70 mV, frequency of 20 Hz, and step potential of 8 mV. Respective background electrolytes (0.12 mol L^{-1} phosphate buffer, pH 7.0) are shown in dashed lines.

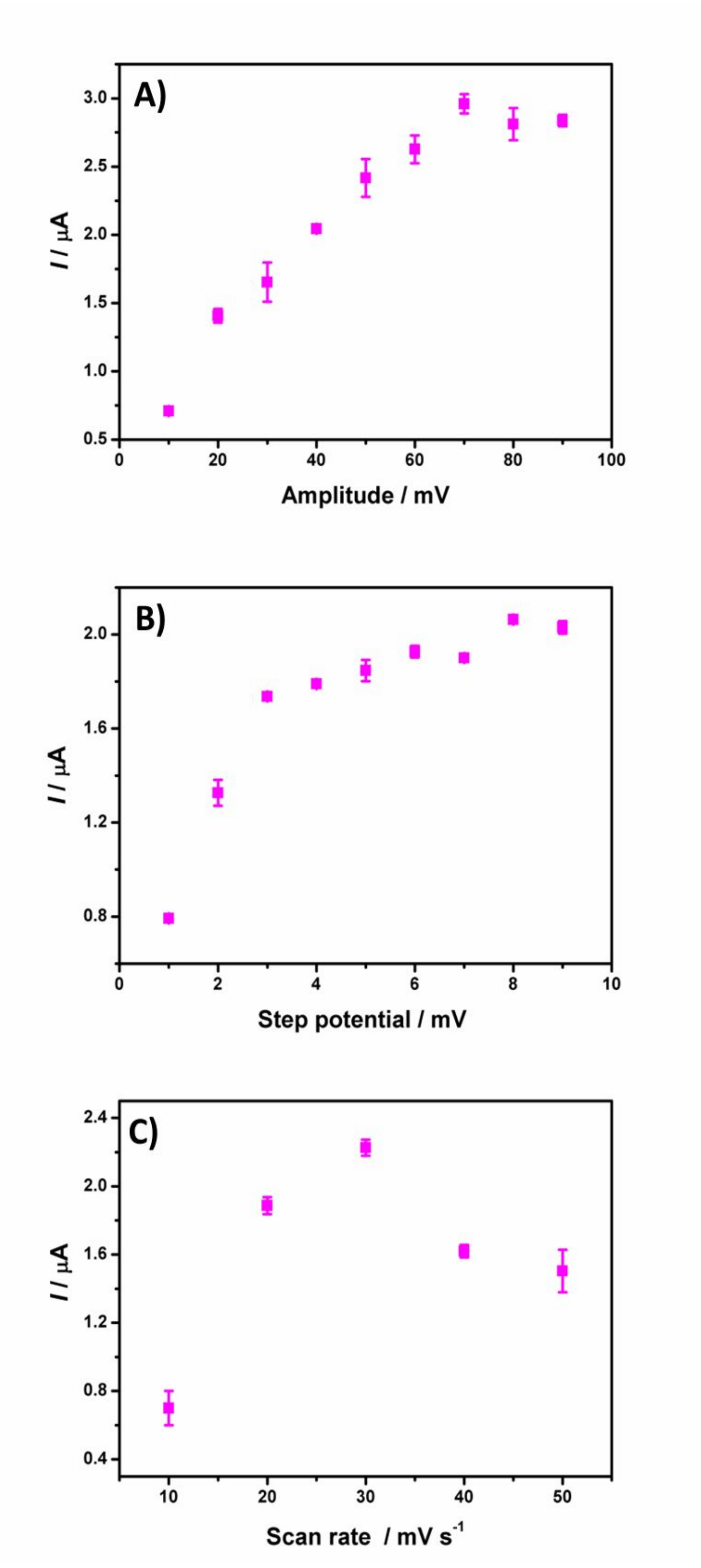

Figure. S8. Impact of DPV parameters (A) modulation amplitude (10 to 90 mV), (B) step potential (1 to 9 mV), and (C) scan rate (10 to 50 mV s^{-1}) on the electrochemical response of 4 µmol L^{-1} 5-HT in 0.12 mol L^{-1} phosphate buffer (pH 7.0) using the 3D-printed G/coumarin-benzothiazole/PLA (30:5:65 *wt.* %) electrode.

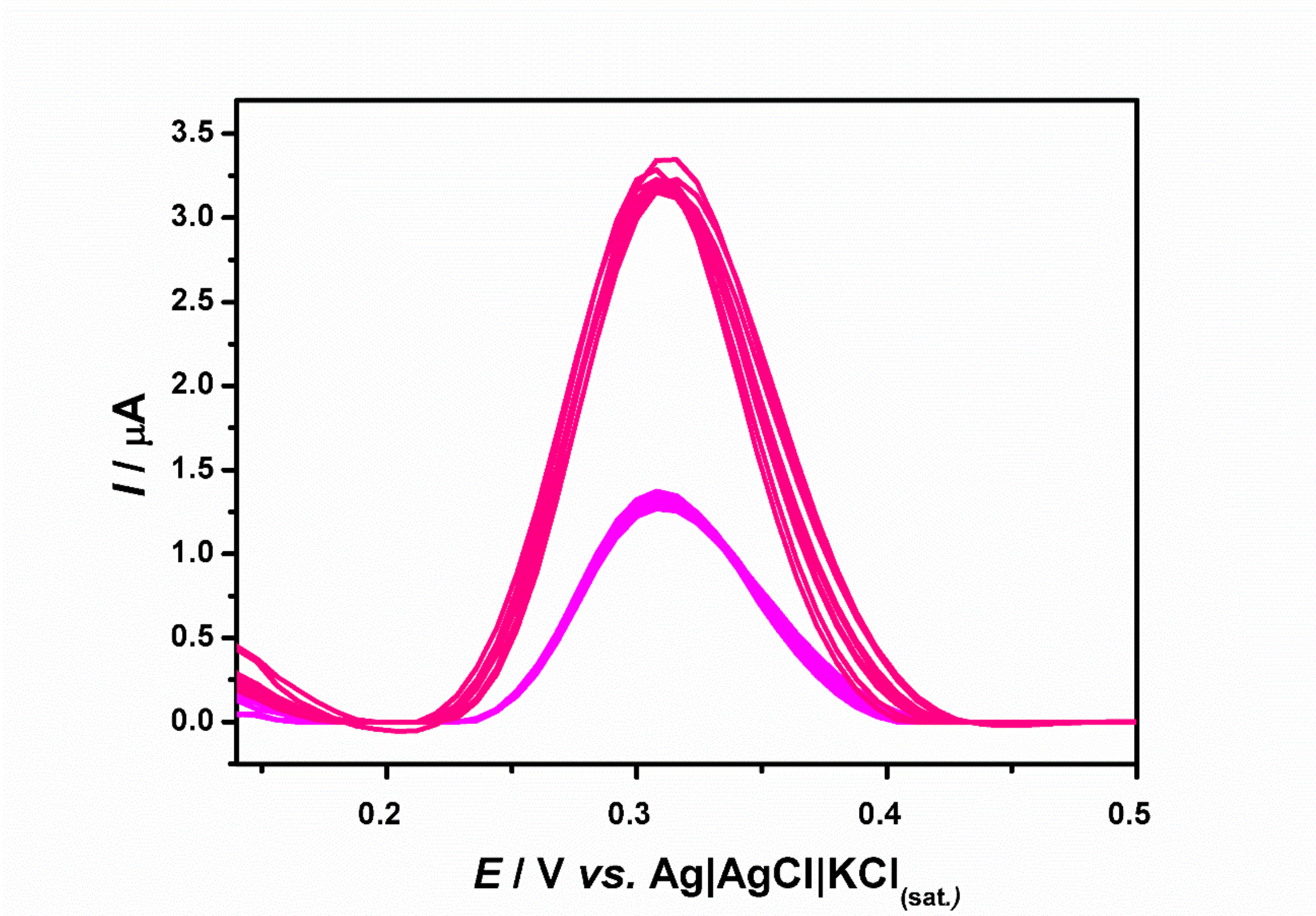

Figure S9. Consecutive DPV voltammograms (n = 8) obtained using two 5–HT concentration levels, 2.5 μmol L^{-1} (magenta lines) and 5.5 μmol L^{-1} (pink lines) upon a 3D-printed G/coumarin-benzothiazole/PLA (30:5:65 *wt.* %) electrode. Supporting electrolyte: 0.12 mol L^{-1} phosphate buffer (pH 7.0). DPV conditions: modulation amplitude of 70 mV, scan rate of 30 mV s^{-1}, and step potential of 8 mV.

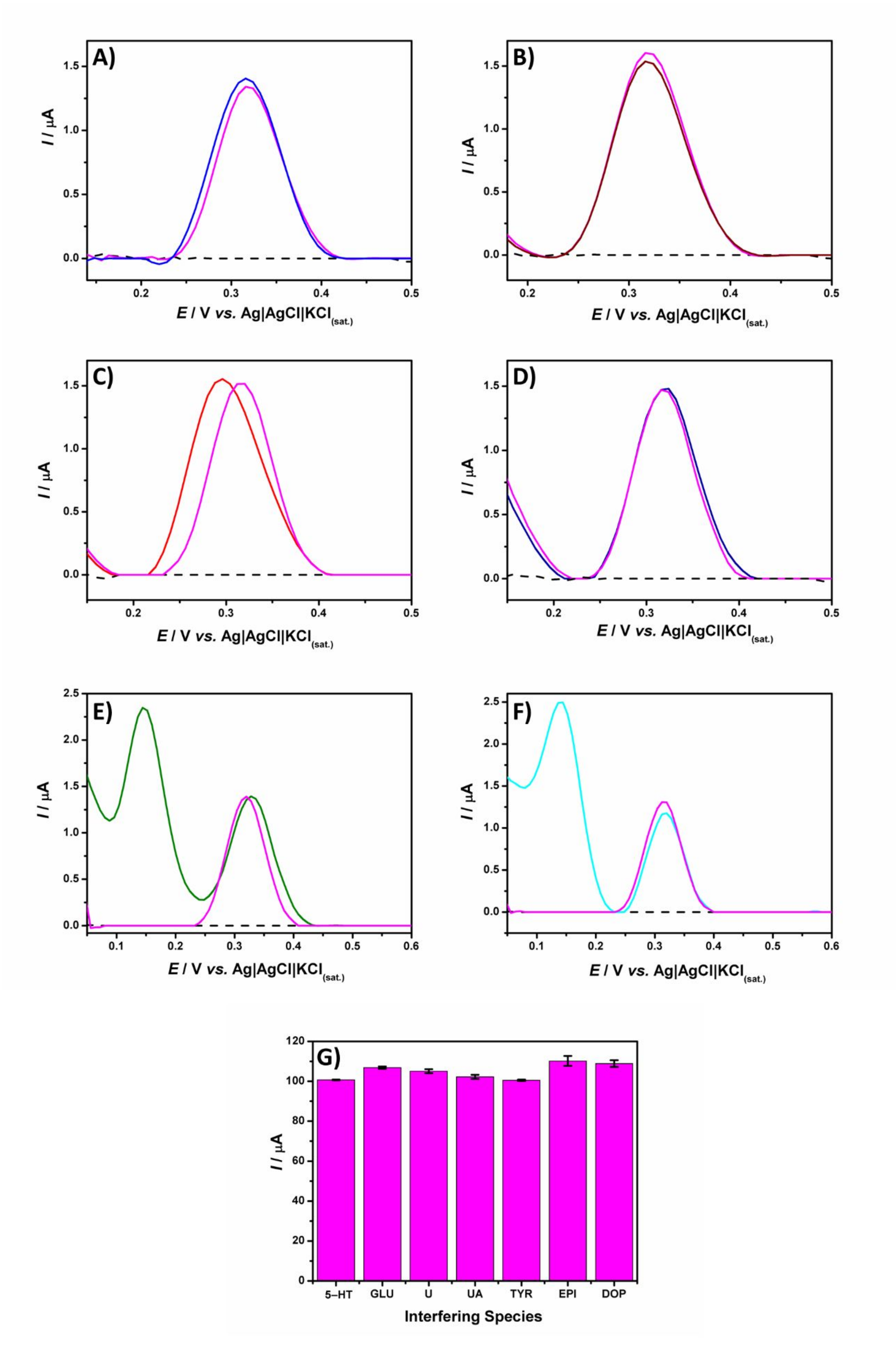
A)
B)
C)
D)
E)
F)
G)
I / µA
E / V vs. Ag|AgCl|KCl(sat.)
5–HT
GLU
U
UA
TYR
EPI
DOP
Interfering Species

Figure S10. DPV responses of 2.5 µmol L^{-1} 5–HT in the absence (magenta lines) and presence of other interferences (A) 8.0 µmol L^{-1} glucose (GLU, blue line), (B) 8.0 µmol L^{-1} of urea (U, brown line), (C) 8.0 µmol L^{-1} of uric acid (UA, red line); (D) 8.0 µmol L^{-1} of tyrosine (TYR, dark blue line); (E) 8.0 µmol L^{-1} of epinephrine (EPI, green line); (F) 8.0 µmol L^{-1} of dopamine (DOP, cyan line); (G) Influence of each interfering compound on the electrochemical response of 5–HT. DPV conditions: modulation amplitude of 70 mV, scan rate of 30 mV s^{-1}, and step potential of 8 mV. Background electrolyte: 0.12 mol L^{-1} phosphate buffer (pH 7.0).

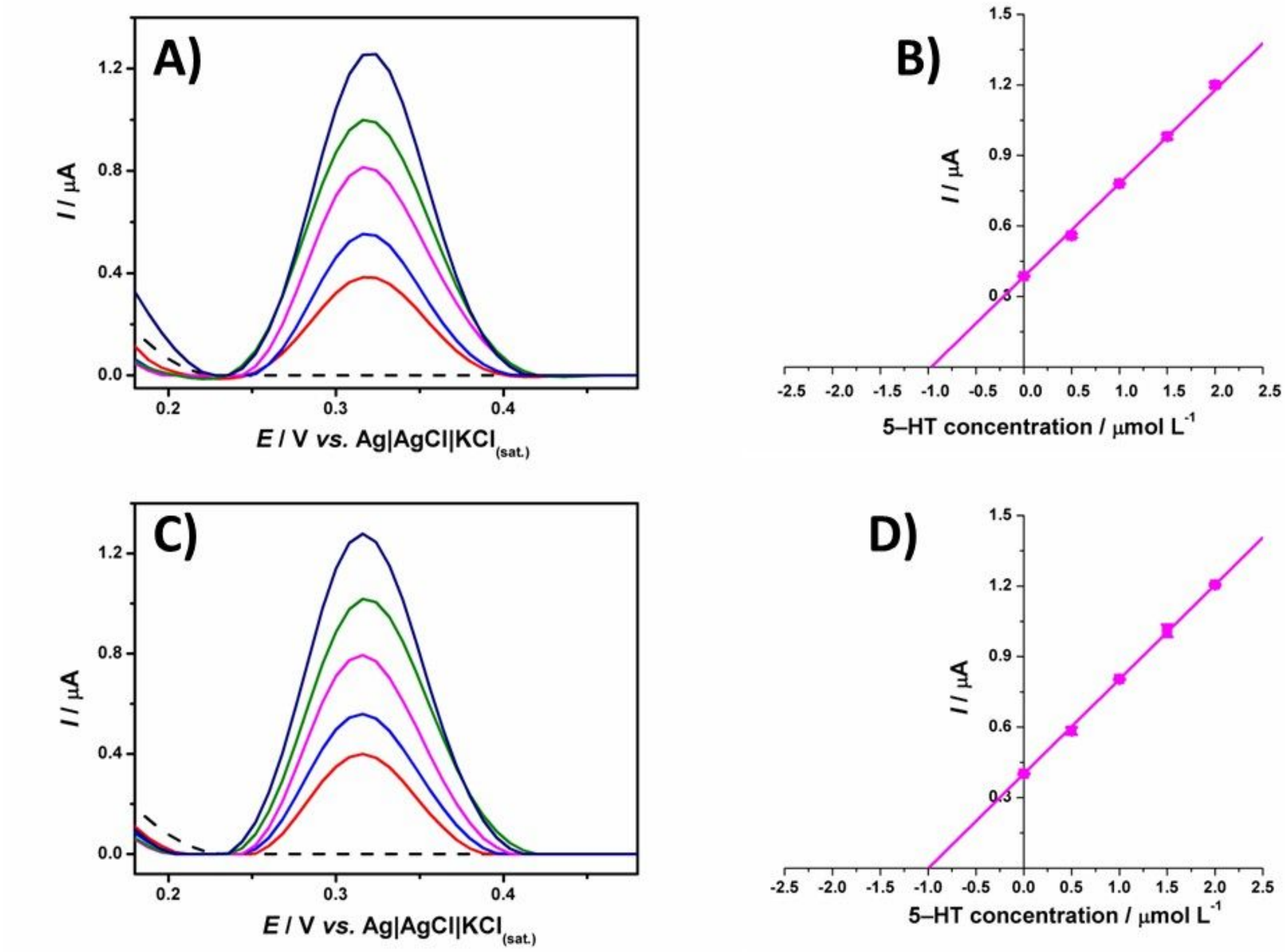

Figure. S11. DPV responses (n = 3) obtained for human serum (A) and cerebrospinal fluid (C) using 3D-printed G/coumarin-benzothiazole/PLA (30:5:65 *wt.* %) electrodes. The respective calibration plots by the standard addition method are represented by B and D. The voltammograms show the non-spiked sample (black lines), and spiked sample at

1.0 μmol L^{-1} 5–HT (red line), followed by the four standard additions (blue, magenta, green, and blue lines). DPV conditions: modulation amplitude of 70 mV, scan rate of 30 mV s^{-1}, and step potential of 8 mV. Background electrolyte: 0.12 mol L^{-1} phosphate buffer (pH 7.0).

Tables

Table S1. Main ATR-FTIR absorption bands identified in the G/PLA and G/coumarin-benzothiazole/PLA electrodes and their corresponding vibrational assignments.

Assignment	Coumarin-benzothiazole	G/PLA (30:70 *wt.* %)	G/coumarin-benzothiazole/PLA (30:2.5:67.5 *wt.* %)	G/coumarin-benzothiazole/PLA (30:5:65 *wt.* %)	G/coumarin-benzothiazole/PLA (30:7.5:62.5 *wt.* %)
**n(O-H) / cm^{-1}**	3350	-	-	-	-
**n(C-H) / cm^{-1}**	3068	2920/2855	2916/2852	-	2916/2853
**n(C=O) / cm^{-1}**	1722	1733	1747	1749	1747
**n(C=C) / cm^{-1}**	1606	1638	1643	-	1636
**n(C=N) / cm^{-1}**	1574	-	-	-	-
**n(C-C) / cm^{-1}**	-	-	1536	-	1541
**n$_a$(C-H/CH$_3$) / cm^{-1}**	-	1455	1449	1448	1448
**n$_s$(CH$_3$) / cm^{-1}**	-	-	1380	1382	1380
**n$_s$(C-H) / cm^{-1}**	-	1350	1353	1353	1350
**n(C-O) / cm^{-1}**	1311	-	-	-	-
**n(C-O-C) / cm^{-1}**	1206/1129	1179/1086	1180/1081	1179/1080	1180/1081
**r(CH$_3$) / cm^{-1}**	-	-	1127	1126	1125
**n$_a$(C-CH$_3$) / cm^{-1}**	-	1031	1038	1040	1038
**n(C-S) / cm^{-1}**	757	-	-	-	-

Table S2. XRD peak parameters of the graphitic (002) reflection in G/PLA and coumarin-benzothiazole-based electrodes.

Sample	2q / º	Intensity/a.u.	d/Å
G/PLA (30:70 *wt.* %)	26.45	2904	3.37
G/coumarin-benzothiazole/PLA (30:2.5:67.5 *wt.* %)	26.65	3311.33	3.34
G/coumarin-benzothiazole/PLA (30:5:65 *wt.* %)	26.35	5244.67	3.38
G/coumarin-benzothiazole/PLA (30:7.5:62.5 *wt.* %)	26.50	3951.33	3.36

Table S3. Raman parameters of G/PLA (control material) and G/coumarin-benzothiazole/PLA electrodes.

Sample	D band/cm^{-1}	G band/cm^{-1}	I_D/I_G
G/PLA (30:70 *wt.* %)	1341.80	1575.74	0.528
G/coumarin-benzothiazole/PLA (30:2.5:67.5 *wt.* %)	1344.07	1575.69	0.253
G/coumarin-benzothiazole/PLA (30:5:65 *wt.* %)	1345.35	1576.13	0.230
G/coumarin-benzothiazole/PLA (30:7.5:62.5 *wt.* %)	1343.68	1575.64	0.368

Table S4. Electrochemical data acquired for 1 mmol L^{-1} $[Fe(CN)_6]^{4-/3-}$ redox probe utilizing G/PLA and G/coumarin-benzothiazole/PLA materials as working electrodes.

**Electrodes**	I_{pc}/µA	I_{pa}/µA	I_{pa}/I_{pc}	ΔE/mV
G/PLA (30:70 *wt.* %)	-8.33	9.55	1.14	0.535
G/coumarin-benzothiazole/PLA (30:2.5:67.5 *wt.* %)	-12.47	11.51	0.95	0.165
G/coumarin-benzothiazole/PLA (30:5:65 *wt.* %)	-25.42	22.5	0.88	0.160
G/coumarin-benzothiazole/PLA (30:7.5:62.5 *wt.* %)	-16.96	19.03	1.12	0.220
